# Assessing direct effects of insect change on insectivore populations in the United Kingdom

**DOI:** 10.1007/s10531-026-03329-5

**Published:** 2026-04-06

**Authors:** L. C. Evans, M. D. Burgess, S. G. Potts, W. E. Kunin, R. Fox, K. E. Powell, K. Boughey, C. A. Harrower, Y. Bourhis, B. Martay, T. H. Oliver

**Affiliations:** 1 School of Biological Sciences, University of Reading, Reading, UK; 2 Butterfly Conservation, Manor Yard, East Lulworth, Wareham, Dorset, UK; 3 RSPB Centre for Conservation Science, Sandy, UK; 4 Centre for Agri-Environmental Research, School of Agriculture, Policy and Development, University of Reading, Reading, UK; 5 School of Biology, University of Leeds, Leeds, UK; 6 Bat Conservation Trust, Quadrant House, 250 Kennington Lane, London, SE11 5RD UK; 7 Centre for Ecology & Hydrology, Crowmarsh Gifford, Wallingford, Oxfordshire UK; 8 Rothamsted Research, Harpenden, UK; 9 British Trust for Ornithology, The Nunnery Thetford, Norfolk, IP24 2PU England; 10 Centre for Ecology and Conservation, University of Exeter, Penryn, United Kingdom; 11 Biodiversity, Land Use and Investment directorate, Defra, London, United Kingdom of Great Britain and Northern Ireland

**Keywords:** Insect declines, Insectivores, Population dynamics, Trophic interactions, Citizen science, Causal inference

## Abstract

Declines in insect abundance are a cause for concern, with potential downstream impacts on ecosystem function. Insects are key food resources for insectivorous vertebrates, with evidence that reductions in prey availability may influence population dynamics. Yet quantifying direct effects at large spatial scales is difficult, both due to data limitations and because correlations may reflect shared responses to environmental change rather than trophic links. We assess the role of insect abundance in the dynamics of 10 insectivores (five birds, five bats) in the United Kingdom by pairing insect and vertebrate abundance data at three spatial resolutions (100, 50, 10 km) from multiple citizen science programmes. To address the challenges of inference and combining heterogeneous data, we adopt a multiple-specification approach spanning: (1) association, (2) prediction, and (3) causal inference. We found evidence of overall declines for all bird species evaluated and for most indices of insect abundance, though none of the bat species tested showed consistent declines. Despite indices of both insect and bird abundance declining, declines did not always co-occur spatially. We also detected only moderate evidence of links between insect change and insectivore population change, notably blue tit and great tit with moth abundance, and grey partridge with Diptera abundance. Across taxa, spatial scales, and analytical approaches, we did not find consistent evidence that changes in insect abundance are a dominant driver of insectivore declines. Both insects and insectivores may instead be responding to multiple, and sometimes non-overlapping, pressures associated with environmental change. We emphasise that absence of strong evidence does not imply absence of effect, and highlight the difficulties of drawing direct conclusions from heterogeneous observational monitoring data.

**Supplementary Information:**

The online version contains supplementary material available at 10.1007/s10531-026-03329-5.

## Introduction

There has long been concern over insect declines (Hallmann et al. 2017; Wagner et al. 2021a, b; Warren et al. 2021) though measuring the extent of the declines is subject to methodological challenges (Didham et al. 2020; Müller et al. 2023). Large-scale insect biodiversity monitoring surveys show sustained declines on average (Wagner et al. 2021a, b), though with variation between taxa and across different habitats (Hallmann et al. 2020; Powell et al. 2023; Wagner et al. 2021a, b). Loss of insect biodiversity is a concern in itself (Clayton 2003; Wilson 1986), but the necessity of understanding the causes and consequences of insect declines is underscored by the wide array of ecosystem functions and services insects provide (Forister et al. 2019; Goulson 2019; van der Sluijs 2020). One major concern relates to the importance of insects in food webs. As insects are important dietary components for many vertebrates, declines in insects could have knock-on repercussions on population dynamics and ecological function. There is some evidence that insect decline may be impacting insectivorous birds (Bowler et al. 2019; Hallmann et al. 2014; Stanton et al. 2018) at least in specific habitats (e.g. farmland), but shared population responses to environmental pressures cannot be ruled out (Pearce-Higgins and Morris 2023).

The role of insect change in insectivore population trends has often been assessed through either indirect approaches, which evaluate the impacts of environmental drivers where effects on insectivores are expected to be partially or fully mediated through changes to insect abundance. For example, Hallmann et al. (2014) found that pesticide use was negatively associated with bird population trends, a pattern plausibly driven in large part by reductions in insect prey, given the strong sensitivity of insects to pesticide exposure. Similarly, Rigal et al. (2023) linked reduced European bird populations to agricultural intensification measured through pesticide and fertiliser use, and comparisons of population trends among species differing in their reliance on insects have suggested insect decline as a potential driver of insectivorous bird declines (Bowler et al. 2019).

However, a key limitation of such indirect approaches is that they estimate the total effect of shared environmental drivers and cannot empirically distinguish effects mediated through insect abundance from direct impacts on insectivores. For instance, pesticides may plausibly reduce insectivores primarily by reducing food availability, but they can also exert direct toxic or sub-lethal effects on birds independent of insect abundance (Molenaar et al. 2024).

An alternative approach, therefore, is to use insect population data to directly assess the effect of insect abundance change on insectivores. However, spatially and temporally overlapping population data from insects and insectivores are limited, making links between insect and insectivore population changes challenging to test. One approach to tackle this constraint is to combine multiple smaller-scale studies through synthesis and meta-analysis (Grames et al. 2023), although here inference is necessarily limited to the species interactions, locations, and times that have been relatively well studied. Alternatively, recent efforts show there is potential to estimate links between insects and insectivores by utilising citizen science monitoring data from standardised recording schemes (Evans et al. 2024; Martay et al. 2023; Yazdanian et al. 2024). While each such scheme targets different taxa, the substantial spatial and temporal replication of some schemes offers the potential to tie together local populations of insects and insectivores to assess direct effects of insect change at regional-to-national scales.

Utilizing national-scale monitoring data, however, brings additional challenges regarding measurement and inference. At the local scale, abundance will be measured with some error and this compounds when tying together monitoring schemes from different locations and when the most relevant life-stage is not recorded (e.g. if adult insects are recorded while the bird species in question mainly predates insect larvae). There are also several decision points when pairing and aggregating insect and insectivore data. The first consideration is spatial scale; foraging behaviour may influence the scale at which effects are detected, as mobile insectivores may be able to overcome local reductions in insect abundance by foraging over greater distances (Oliver et al. 2010). This may result in correlations that are weaker at the local scale and stronger at a regional scale. However, aggregating data across different scales can also have non-trivial impacts on signal-to-noise ratios and statistical power. Second, insectivores are likely to eat multiple insect species; therefore, there is a need to generate appropriate indices of overall food abundance from species-level data. Such indices could be aggregated under different weighting schemes (i.e. varying species importance) and with different units (e.g. abundance, biomass; Anthony & Kunz, 1977; Naef-Daenzer et al., 1999).

A final consideration when using national-scale monitoring data is confounding effects, as correlated population fluctuations between insects and insectivores may result from shared responses to changing environmental variables (e.g. weather, habitat or land cover change). Omitting or lacking control for such factors can bias estimates of the role of insects, potentially either over- or underestimating their impact. Additionally, even if direct effects are present, tightly coupled population dynamics between predators and prey can result in positive, negative, or zero correlations in abundances over time (Sugihara et al. 2012), resulting in shifting variable importance in a linear statistical analysis.

These issues around complex causal structures, confounding, data quality, and model specifications risk incurring both Type I and Type II errors, as, (1) we may falsely identify a link between insects and insectivore dynamics that could be due to noise or confounding variables, particularly shared responses to environmental variation; or, (2) we may fail to detect a link when there is one, due to measurement error (which will bias coefficients towards zero), improper controls, or model specifications that are unable to capture dynamic features.

Given the challenges both in terms of varying data quality and causal uncertainty, we think it is useful to analyse the role of insects on insectivore dynamics through a combination of approaches rather than any single analysis, especially given that researchers can generate different conclusions using the same data (Gould et al. 2023). We take inspiration from specification approaches (Simonsohn et al. 2019) by providing several tests evaluating links between insects and insectivores.

Our approach is to follow simple associative tests – which can help identify basic patterns in the data – to non-linear predictive approaches, useful for capturing associations that linear methods may be unable to identify, before using linear ‘causal inference’ models to help account for confounding.

The associative tests evaluate basic patterns between the dynamics of the insects and the insectivores, i.e. are the directions of long-term population trends similar across space? While any correlations between the trends could obviously not be considered causal, they are still informative. For example, if both insectivores and insects are declining, but the declines are in different locations, then we can lower our confidence that insect declines are driving insectivore declines. Therefore, while the approaches below provide better control for confounding, they might obscure these basic patterns (particularly after controlling for variation associated with site, year, or spatial factors).

Next, we use Empirical Dynamic Modeling (EDM) to test if insect abundances can predict insectivore dynamics (i.e. Granger Causality; Granger, 1969). This approach, while not free from spurious associations due to confounding, can capture temporal lags in associations and shifting correlations due to coupled dynamics that can be missed in linear statistical approaches. For example, if this approach showed that insects were highly predictive of insectivore dynamics, but linear approaches found no association, then it may provide grounds to explore factors such as tightly coupled population (i.e. top-down and bottom-up controls), or lagged effects, rather than concluding there is no evidence for insect impacts and insectivore declines.

Finally, we apply linear ‘causal inference’ approaches that, through different means, aim to robustly control for the confounding effects of potential static (e.g. habitat quality) and dynamic factors (e.g. shared responses to weather) that can cause mirage associations between insect and insectivore dynamics. The fixed-effects panel estimator models used are designed to minimise the risk of Type I errors by accounting for unobserved heterogeneity and correlated external drivers. However, increased control comes with a trade-off in interpretability and a potential increased risk for Type II errors for certain specifications of these models.

Using our specification approach and assessing evidence holistically across different methods, we evaluate evidence for the role of changing insect abundance in insectivore decline for several insect groups and 10 insectivorous vertebrate species. Specifically, we ask three main questions: (1) Are insect and insectivores’ long-term trends and interannual changes correlated across space? (2) Does information on insect abundance predict insectivore abundance change? (3) After controlling for shared environmental factors, is there evidence for a direct effect of insects on insectivore dynamics?

## Methods

### Insectivore data

Data for insectivores were derived from the Breeding Bird Survey (BBS; https://www.bto.org/our-science/projects/breeding-bird-survey, Massimino et al. 2025) and the National Bat Monitoring Programme (NBMP; https://www.bats.org.uk/our-work/national-bat-monitoring-programme, Barlow et al. 2015). Species were selected to span a range of habitat associations, dietary reliance on different insects, and variation in overall abundance.

For the BBS, volunteers walk two transects across a 1 km square twice a year during the bird breeding season with observations organised into four distance categories (0–25, 25–100, > 100 m and flying over). To generate indices of relative local abundance, we summed observations from all categories for each visit (other than those ‘flying over’, as these birds may not be members of the local breeding population) and took the maximum number observed during a visit as the index of abundance. We selected five insectivorous species that occupy a range of habitats and which specialise on different insect groups: great tit (*Parus major*), blue tit (*Cyanistes caeruleus*), grey partridge (*Perdix perdix*), skylark (*Alauda arvensis*), and corn bunting (*Emberiza calandra*). For the skylark, we additionally retained ‘flying over’ counts as skylarks sing directly over their breeding territories. Although all these species are insectivorous, they vary in the specificity of the insect prey they target, their overall abundance, and the importance of insects in their overall diet. Therefore, it should be noted that we have not selected for the bird species most likely to be impacted by insects; rather, we provide a selection of species that vary in habitat specialism, traits etc., to attempt to explore the potential importance of insect change for UK birds more generally.

For bats, we used relative abundance data from the NBMP Field and Waterway Surveys. The surveys are standardised for transect length and selected using random stratification. The Field Survey captures the number of ‘passes’ by common and soprano pipistrelle (*Pipistrellus pipistrellus*,* P. pygmaeus*) at point counts along a field transect, and by noctule (*Nyctalus noctule*), and serotine (*Eptesicus serotinus*) along the walked section between each point. The Waterway Survey captures the number of Daubenton’s bat (*Myotis daubentonii*) passes at point counts along a transect adjacent to a waterway.

For both schemes, we generated relative indices of site-level abundance by summing the passes across all transect sections observed during a visit and then selecting the maximum observed as the yearly index. We additionally only used data from completed transects and removed sites where the species was never observed. One additional challenge with these data is over-dispersion in passes due to the potential for counting the same individuals more than once. Previous approaches have circumvented such issues by using alternative measures of abundance, such as the number of transect sections the bats were found to occupy (Barlow et al. 2015). However, this approach removes information and adds an upper bound to the index. As we were interested in indices of relative abundance, we treated the potential for over-dispersion as a source of additional noise that we expected to be consistent across years, but we note that the bat indices are likely to have more noise relative to our bird and insect indices.

### Insect data

We assembled data from four insect monitoring programmes that provide indices of relative abundance for moths, butterflies, freshwater invertebrates, and carabid beetles. Schemes for butterflies, moths, and freshwater invertebrates provide a relatively high spatial coverage at either the country or national level (England), whereas data for the beetles were available for 12 sites. We derived indices at the order level for all groups except carabids, which are necessarily at the family level, and we kept moths and butterflies separate as they are recorded in different schemes. We chose to use combined indices at the order and family level to constrain the already extensive analysis, but also because insectivores are expected to eat a variety of insect prey within an order and previous research indicates a signal of insect abundance on insectivore dynamics at the order level (Evans et al. 2024) or with combined insect indices (Martay et al. 2023). A combined index might be biased towards more detectable rather than abundant species, but there was not sufficient information on inter-specific variation in detectability to control for such effects across our insect taxa.

Data on moth abundance were derived from the Rothamsted Insect Survey light-trap network (https://www.rothamsted.ac.uk/national-capability/the-insect-survey). The traps currently operate nightly at approximately 80 sites. For a local index of population abundance, we used the site-level indices produced by Harrower et al. (2020) covering the period 1968–2017. These data comprise species-level indices of abundance that we subsequently converted into order-level indices by summing the relevant species-level indices for each site and year.

The butterfly indices were derived from the UKBMS 2019 site-level indices (Botham et al. 2020). These indices are measures of relative abundance calculated using standardised methods from the data collected in the UK Butterfly Monitoring Scheme (https://ukbms.org/; Dennis et al. 2016) spanning the period 1976–2019.

Abundance data for freshwater invertebrates were extracted from the Environment Agency’s ecological monitoring database, which covers rivers in England (Environment Agency, 2020). We used the pre-processing applied by Powell et al. (2023) to derive indices of local abundance by summing the abundances derived from 3-minute kick-samples.

Data for carabid beetles were derived from carabid beetle surveys undertaken at the terrestrial sites of the Environmental Change Network (Rennie et al. 2017). The surveys consist of standardised deployment of pitfall traps at twelve sites undertaken throughout the year across the period 1992–2015. Unlike the other insect data here, we derived our own indices by estimating fixed effects of yearly abundance. Details of our approach are presented in the supplementary materials.

### Weather data

For comparisons of the predictive role of insects relative to weather, we used the HadUK gridded 5 km observations (Met Office et al. 2023), which provide observations of annual and seasonal mean temperatures and total precipitation at the 5 km scale (https://www.metoffice.gov.uk/research/climate/maps-and-data/data/haduk-grid/overview*).* We averaged these values to scale to relevant grid sizes (see below).

### Pairing insects and insectivores

To select the candidate insect food source(s) we used a combination of expert opinion and literature review. Our approach was to fill matrices of potential food sources (insectivores-by-insect) using three categories: (1) insect taxon is a primary food source, (2) insect taxon is a secondary food source, or (3) limited or no evidence of the insect taxon as a food source. The matrices were at the level of insect families nested within orders. The categorisation of the birds was undertaken by a taxon expert (*MB*), supported by the relevant literature. For the bats, the importance categories were conducted by *LE* informed by a rapid review of the diet literature for bats. The matrices and references supporting the assessments are presented in the code and data supplement (10.5281/zenodo.15037981).

After categorising food importance for our 10 species, we cross-referenced the selections with the insect data, aiming to identify one or two primary candidates for the analysis. Occasionally, a key resource was not tested due to data limitations. For example, diet studies for noctule and serotine bats in the UK highlight *Scarabaeoidea* as a key resource; however, we did not have data for this beetle family. Similarly, carabid beetles are taken by grey partridge; however, we had insufficient overlap between the data sets to test associations. For Diptera (true flies), we only utilised data for aquatic species (i.e. aquatic larvae), though we note most species will be primarily feeding on the adults.

Our selections are as follows: we paired blue tit and great tit with moths, corn bunting with moths and butterflies, skylark with *Carabidae*, and grey partridge with aquatic Diptera. For bats, we tested common and soprano pipistrelle with aquatic Diptera, noctule with aquatic Diptera and moths, serotine with aquatic Diptera and moths, and Daubenton’s bat with aquatic Diptera.

### Aggregating and pairing by grid squares

**Fig. 1:**
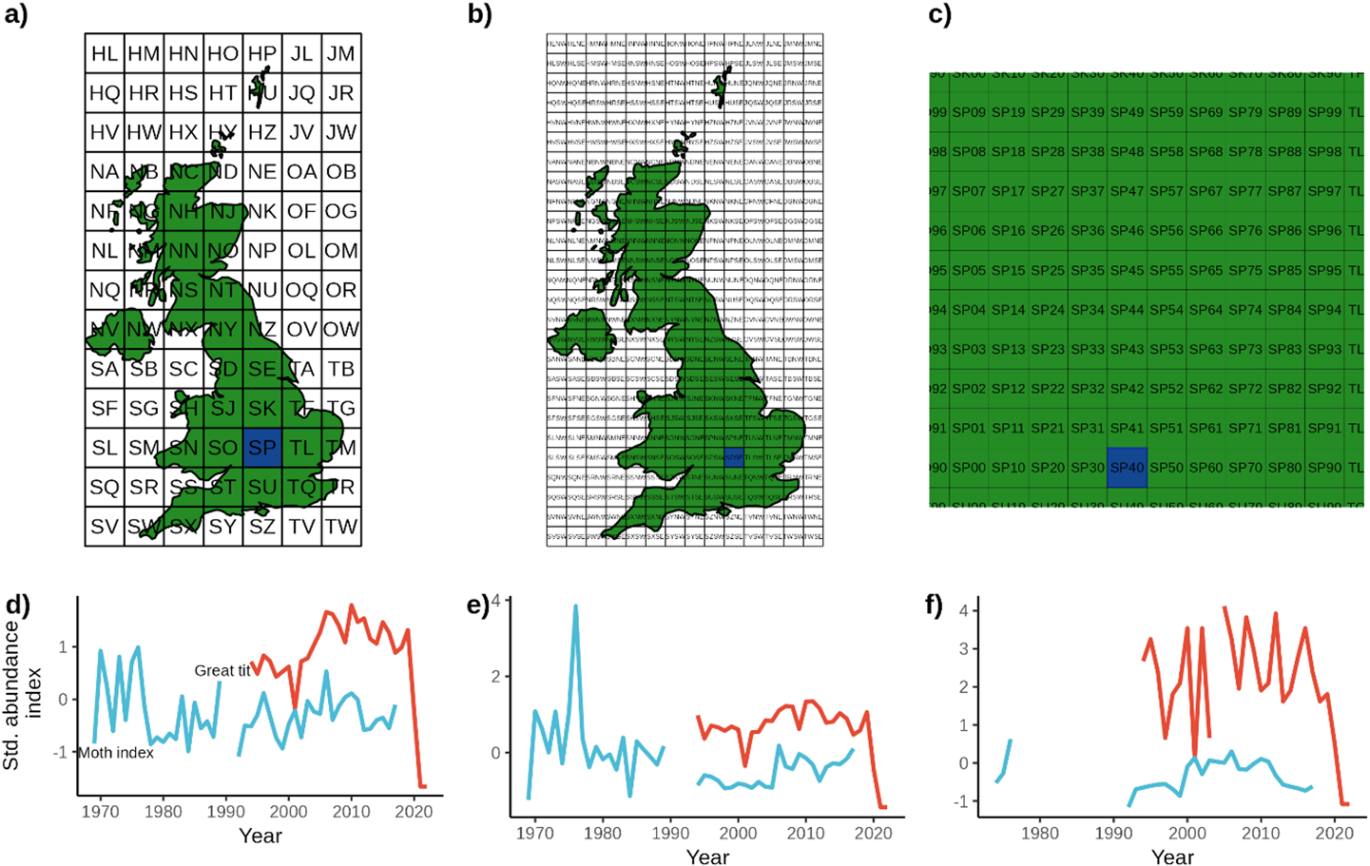
Demonstration of the grid-based strategy: (**a**) 100 km, (**b**) 50 km, and (**c**) 10 km grid squares. **d**), **e**), and **f**) show the generated standardised abundances for the great tit (red line) and moth index (blue line) for a selected (and nested) grid square at each scale. Grid squares vary in coverage across space and time, with the indices becoming sparser at higher spatial resolutions

To combine and pair insect, insectivore, and weather data, we used a grid-based approach using the Ordnance Survey national grid reference system. To generate grid-level indices, we averaged, for each year, the data for each insect, insectivore, and weather index at three scales: 100 km, 50 km and 10 km grid squares. This resulted in a time series of relative abundance for each taxon at the different spatial aggregations. Insect-insectivore time series were then paired within each grid square to test for the impact of insect abundance on insectivore dynamics (Fig. 1).

Our grid-based approach is one of several potential methods for linking proximate surveying sites from the different monitoring schemes. We favoured this approach for several reasons. First, it provides a simple and objective method to tie together indices at fixed levels of aggregation without concern for utilising the same data in multiple comparisons. Second, it’s a well-utilised reference system that situates the trends and dynamics of the target species within a recognisable spatial context. Finally, we take advantage of the nesting structure of the national grid system with smaller spatial units within larger ones to correlate standard errors in the fixed effects panel estimators (see below). A downside of the grid-based approach is that there might be sites near the edges of grids that are closer in geographic space, but which are nevertheless assessed in different pairings; however, we view this as a reasonable trade-off given the described advantages.

### Overall analytic strategy

We used three approaches: (1) *association*, (2) *prediction*, and (3) *causal inference* to test the main hypotheses. For *association (1)*, we used Seasonal and Trend decomposition using Loess (STL; Cleveland et al. 1990), which partitions a time series into a trend and remainder. The trend captures the long-term trajectory of the population, and the remainder captures inter-annual fluctuations. For *prediction (2)*, we used Empirical Dynamic Modelling (specifically, Gaussian process regression EDM; Munch and Rogers 2024). This method can assess potential links (including non-linear and time-varying interactions) between insects and insectivores by demonstrating how prediction accuracy increases with the inclusion of certain variables (i.e. Granger causality, Granger, 1969; Shojaie & Fox, 2022). Finally, for *causal inference (3)*, we used a panel of fixed effects estimators (Wooldridge 2010). These tested for linear interactions after controlling for static grid-level average differences in population growth and shared dynamic effects (i.e. shared yearly environmental effects), while utilising clustered standard errors to capture correlated errors within regions (i.e. 100 km squares).

### Association (1): STL decomposition

We decomposed each time series into a long-term trend and remainder (interannual change beyond the trend) by applying STL at all three spatial scales for time series with five or more years of consecutive abundance data. We used STL as it splits time-series into trends and remainders, which allows us to separately evaluate if the trends in insectivores and insects are correlated across space or if interannual changes beyond the trend are correlated (suggestive of either direct effect or shared environmental responses). Because data are annual/seasonally restricted, STL here functions primarily as a smoother separating long-term variation from interannual deviations (similar to LOESS + residual), rather than estimating within-year seasonality. As STL cannot incorporate missing data, we took the longest consecutive run of data at each spatial scale, keeping only those grids where there were at least ten years of data, excluding sites where > 50% of the time series was zero, as such sites provide little information on trend. To assess spatial coherence in trend and short-term deviations, we correlated the STL trend and remainder components across space. Specifically, for the remainders for each insect–insectivore pairing and year, we computed Pearson correlations between the vectors of remainder values across all pairs of grid cells (i.e. grid–grid correlations), and summarised the resulting distribution of correlations. This assessed whether local departures from long-term trends tend to co-occur spatially across taxa. For the trend, we initially fitted a mixed linear model for each species with abundance as the dependent variable and year fitted as a continuous variable, including random slopes and intercepts for each grid square. We then took the values of these slopes for insect and insectivore and split them into two categories: increasing or decreasing. We then assessed if there was more agreement in the direction of these slopes than expected by chance through a binomial test. We summarise trends by their direction rather than magnitude to reduce sensitivity to noise in short or irregular time series and to focus on broad spatial agreement rather than local effect size estimates. The STL-based analyses are, therefore, intended as descriptive diagnostics of spatial and temporal patterning, rather than as formal inferential models. We focus only on the direction and consistency of patterns, rather than on effect sizes or uncertainty estimates, which are addressed in the subsequent analyses.

### Prediction (2): gaussian process regression empirical dynamic modelling

Our framework for EDM was Gaussian-process empirical dynamic modelling with automatic relevance determination, applied through the GPEDM package (Munch and Rogers 2024). This hierarchical approach utilises time-delay embedding and shared information across spatial replicates to construct approximations of the state-space manifold, thereby capturing the system dynamics alongside estimating the underlying dynamic correlation, i.e. the similarity in the underlying dynamics across sites. An accessible overview of the EDM approaches is provided by Chang et al. (2017) and Edwards et al. (2024).

We used a time-delay of one year, and to constrain the complexity of the models, a maximum embedding dimension of three years; embedding referring to reconstructing system state using time-lagged values of the focal variable(s) (i.e. Takens’ theorem). To assess the role of insect abundance in insectivore dynamics, we fitted 10 GPEDM models for each insectivore-insect pairing at each scale. The 10 comparisons consisted of five head-to-head comparisons (two models per comparison): (1) insectivore only vs. insectivore and insect; (2) insectivore and spring weather variables vs. insectivore, insect, and spring weather variables; (3) insectivore and summer weather variables vs. insectivore, insect, and summer weather variables; (4) insectivore and winter weather variables vs. insectivore, insect, and winter weather variables; and (5) insectivore and annual weather variables vs. insectivore, insect, and annual weather variables.

Out-of-sample predictive performance was estimated using leave-one-timepoint-out validation. To ensure that lagged states represented one-year steps and to handle missing observations, we retained for each grid square the longest run of consecutive calendar years for the variables included in a given model, retaining the original year index rather than reindexing series to a common time scale.

### Causal inference (3): fixed effects panel estimator

The fixed effects panel estimator is a regression-based approach that attempts to estimate ‘causal’ effects by controlling for static (site-level) and dynamic confounding (year-level) effects. These approaches can be preferable to random effects models for causal inference as they make no distributional assumptions about variation across units and induce no shrinkage; they also make no assumptions of independence between the site-level characteristics and independent variables and are unbiased when the independent variables are correlated with unobserved heterogeneity – factors expected to be common in observational data (Byrnes and Dee 2025). However, estimation differs, as rather than modelling site and year-level variation as draws from a normal distribution, the static and dynamic effects are eliminated through demeaning the independent and dependent variables prior to the regression, e.g. centering the independent and dependent variables at each site. When only site (unit-level) fixed effects are included, we estimate how within-unit deviations from the site mean are influenced by the independent variables. When only time effects are included, the coefficients for the independent variables represent the average (across all years) of how differences in the independent variables between sites within a given year are associated with differences in the dependent variable across sites in that same year. But when both site and time level effects are included, the coefficient of interest represents a complex combination of within-year and across-unit contrasts, which is both challenging to interpret and can generate bias under certain circumstances (De Chaisemartin and d’Haultfoeuille 2020; Goodman-Bacon 2021; Kropko and Kubinec 2020). However, including both site and year fixed effects provides robust control for shared ‘shocks’ (insectivores and insects responding similarly in a given year due to unmeasured confounding factors, e.g. a drought). Therefore, in line with our overall specification approach, we specify a set of different models that vary in controls, generating results that can be interpreted holistically.

The first model was a one-way fixed effect model, with a fixed effect at the unit level (each grid square at a given scale). This model aims to control for unmeasured time-invariant unit-level confounds (e.g. habitat) and estimates the within-unit effect of insect abundance on insectivore growth rate. However, this model provides limited control for possible time-varying factors/shocks (such as shared responses of insects and insectivores to extreme weather). For the 10 km and 50 km scales, we included some control for time-varying factors through clustered standard errors at the regional level (100 km), which account for spatially structured environmental shocks that may induce correlations in the residuals of nearby units. The second model was a two-way fixed effects model including an additional fixed effect at each year that aimed to additionally control for time-varying shocks. But as stated above, we are asking an unusual question: *in a given year*,* do sites with more insect abundance than other sites experience higher population growth relative to the average at these sites?* The final two models had the same fixed effect structures as the above two models, but additionally include covariates for annual temperature and precipitation. Here, for the one-way fixed effect, this attempts to adjust for the shared responses to climate, which, if a main determinant of shared annual responses across sites, should provide better estimates of the impact of changing insect abundance on insectivores. However, these models will not control for all unmeasured temporal factors, especially those uncorrelated with the climatic variables. And finally, the two-way fixed effect has the same benefits and limitations as above, but additionally controls for the effect of shared climate.

The models were instantiated within a linearised Gompertz equation (Eq. 1) predicting the log growth rate – the log ratio of the current and previous years’ population size.

  

1 $$\:\mathrm{log}\left(\varDelta\:{Insectivore}_{it}\right)=\mathrm{log}\left({Insectivore}_{i\left(t-1\right)}\right)+\:{Site}_{i}+{Year}_{t}+{Insect}_{it}+\:{Insect}_{i(t-1)}+{\epsilon\:}_{ir}$$

With *i* relating to each unit (10 km/50km/100km grid square) and *t* to each year. In the panel estimator, standard errors were clustered by the unit (*i)* at 100 km and region (*r*) and unit for the 50 and 10 km models. In this equation, we show the framework for the 50–10 km model, including the fixed effects for year (Year_t_) - though this fixed effect is not present in models 1 and 3. As we were working with log growth rates, we needed to deal with zero indices, as they result in undefined growth rates. We first removed sites for the insectivores that contained more than 50% zeros, as they induce considerable dilution in assessing relationships to insect abundance. For the remaining sites, we computed growth as the change in log(𝑦+1) between consecutive years. This transformation prevents the undefined growth rates and retains observations where populations crash to, or recover from, zero, which are ecologically meaningful events. However, we recognise that adding one alters the scale of the growth rate and may disproportionately affect low counts, but we accepted this trade-off to capture important losses and gains in the abundance indices. All models were fitted using the *feols* function in the *fixest* package (Bergé 2018).

All analysis was undertaken in R 4.4.2 (R Core Team, 2024) with the code and data in support of the results available at 10.5281/zenodo.15037981.

## Results

### Association: correlations between trends and dynamics

**Fig. 2:**
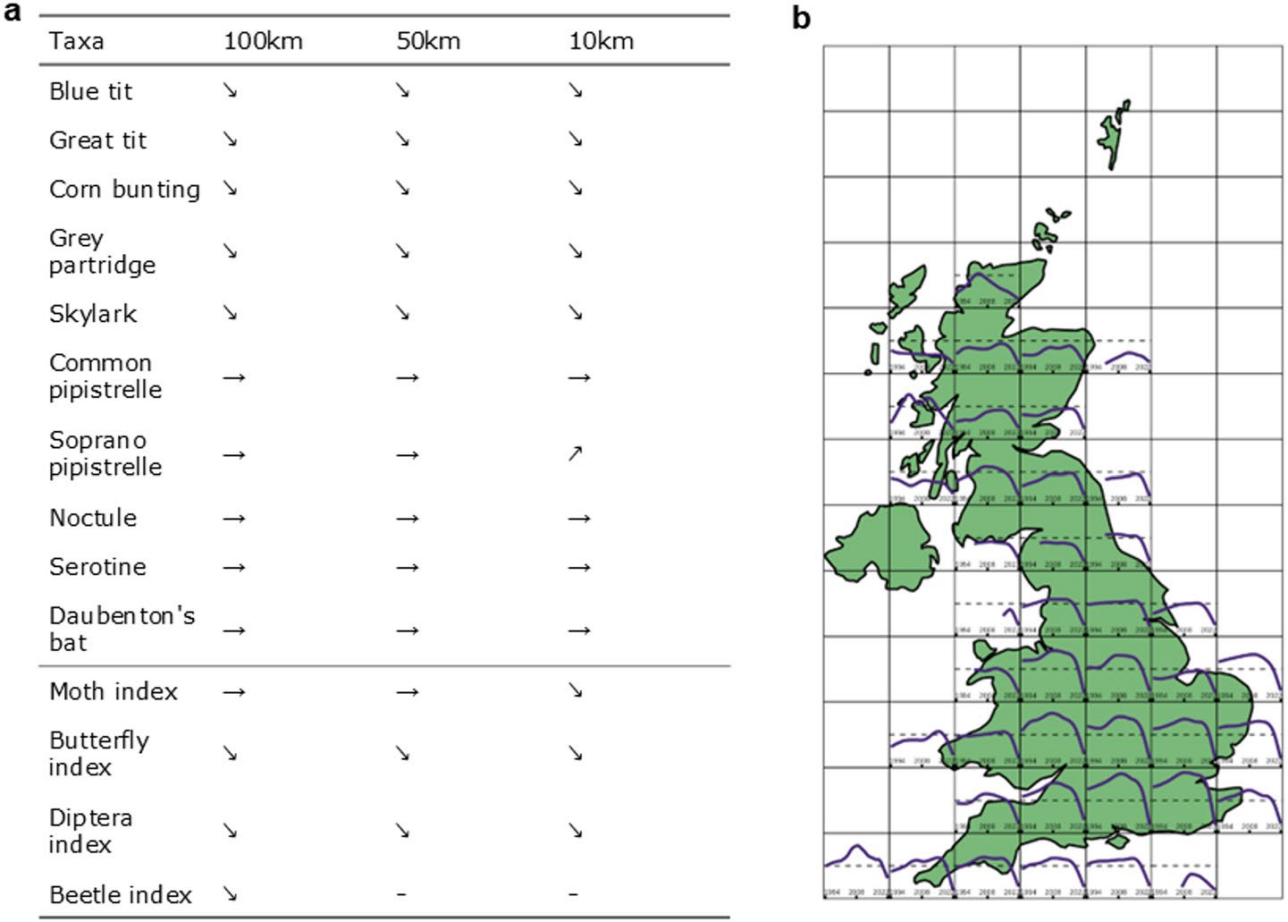
(**a**) Summary of average linear trends for insectivores and insect indices at different grid aggregations. Positive trends are indicated by ↗, negative trends are indicated by ↘, and no change is shown by →. Scales without data presented are indicated by “-“. (**b**) Trends across space for the great tit at 100 km, with dashed lines showing mean abundance across all sites and time points

All birds showed evidence of decline across all scales, but none of the bat species showed evidence of overall decline (Fig. 2).

For indices of insect abundance, we found consistent negative trends across all scales, with only the moth index showing no change at 100 km and 50 km, but negative trends at the 10 km scale.

**Fig. 3:**
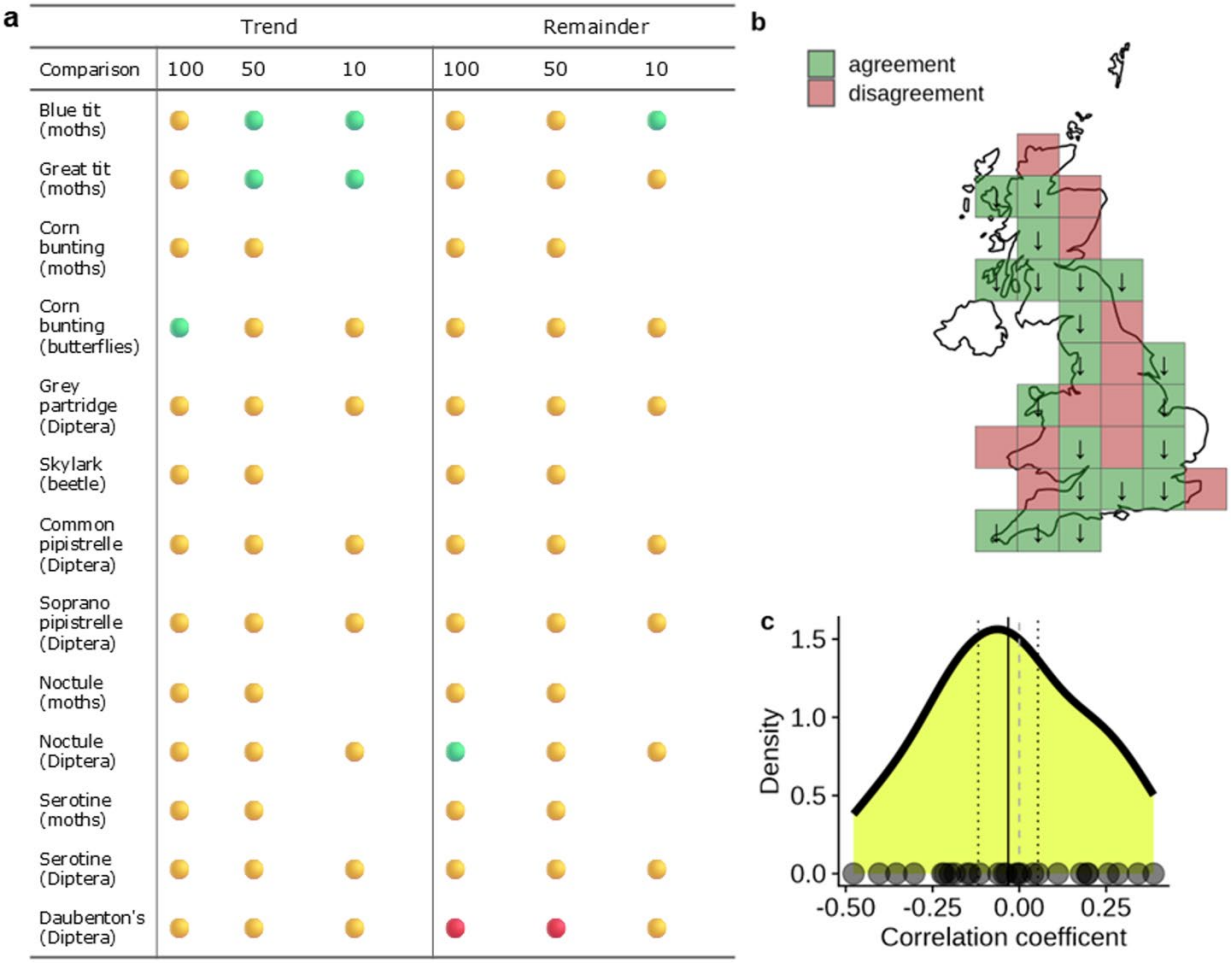
Associations between trends and the remainder (interannual change) for insectivore-insect pairs. (**a**) Correlations between insectivores and food indices for trends and remainders across scales. The green points represent positive associations, the red points negative associations, and the yellow points uncertain associations. (**b**) Shows how trends for the great tit and the moth index compare at the 100 km scale, with locations where the trends have the same sign shown in green and locations where trends have the opposite sign in red. The arrows within each grid square indicate the trend direction where there is agreement. (**c**) Distribution of correlations between the remainders within 100 km grid squares for the great tit and moth index. The solid black line shows the mean correlation, dotted lines show 95% confidence intervals, and a grey dashed line indicates zero

There were few simple associations between the trends in insects and insectivores, though both the great tit and blue tit showed positive correlations with moths at the 50 and 10 km scales, and there was a positive association between butterflies and the corn bunting at the 100 km scale (Fig. 3). For inter-annual change, we found positive associations for blue tit and moths, and noctule and Diptera at the 10 km scale, and negative associations between Daubenton’s bat and Diptera at the 100 and 50 km scale.

### Prediction: empirical dynamic modelling

**Fig. 4:**
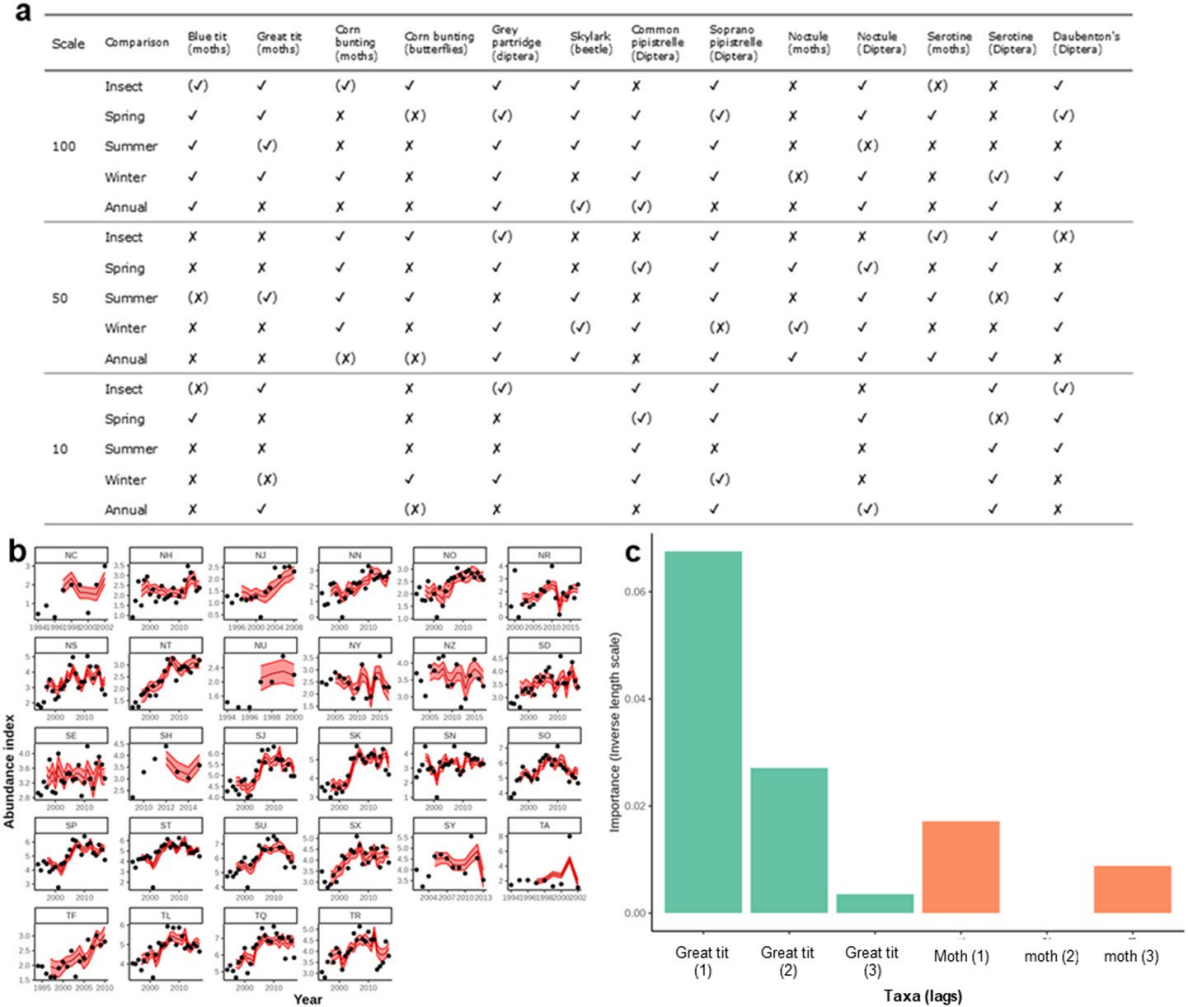
**a**). Model comparisons for EDM, for each comparison ✘ indicates that the model without insects had the highest R^2^, ✔ the insect model had the highest R^2,^ and the symbol in brackets was the single best model at that scale. Example results for great tit at 100 km scale for the insect and insectivore model with **b**) time series of abundance for a selection of grid-squares with the EDM predictions shown in red and observed abundance index in black; **c**) inverse-length scale from the auto relevance determination reflecting the inferred importance of each time-lagged input on predictive performance; lags shown in brackets

Predictive skill generally increased when including insect indices, although with considerable variability across scales and controls (Fig. 4). Improvements in predictive skill were also typically small, and overall performance degraded at smaller scales. For six insectivore species (great tit, grey partridge, skylark, common pipistrelle, noctule and Daubenton’s bat) the majority of the best models across different scales included the insect data. The only species where none of the best models included the insect variable was the corn bunting regarding butterflies.

### Causal links: fixed effects panel estimator

**Fig. 5:**
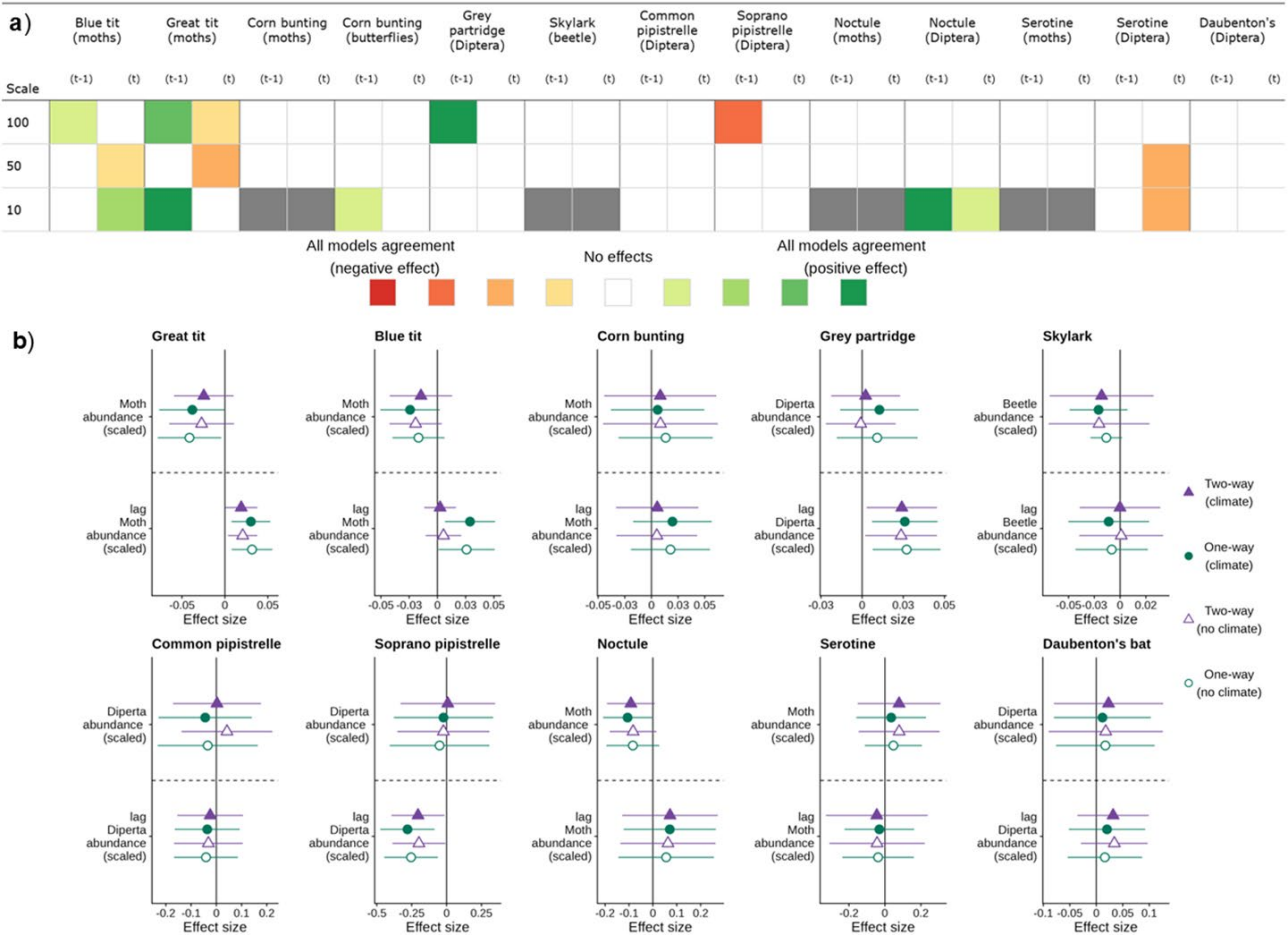
(**a**) Summary of results from the fixed effects panel estimators. The colours (red or green hues) represent the direction of the effect, while the strength of the hue shows the number of models where a significant effect was detected; a grey square indicates the analysis was not conducted at this scale. A positive effect indicates that increases in insect abundance lead to larger growth rates for the insectivore. (**b**) Forest plots of the effect of contemporaneous (above) and lagged (below) insect abundances on population change for comparisons at the 100 km scale

For the fixed effects panel estimators, evidence for direct effects of insects on insectivores was mixed (Fig. 5). Positive links included those for great tit, blue tit, common pipistrelle, grey partridge and noctule, but this varied between model specification and scale, with sometimes the lag or sometimes the concurrent measure of insect abundance showing the effect (e.g. for the blue tit). We also identified negative links for the serotine, soprano pipistrelle and blue and great tit for the concurrent index abundance at some scales. In general, where effects were detected, they were for model specifications 1 and 3 that included only unit-level fixed effects and climate controls.

## Discussion

We have provided a broad assessment of the impacts of insect abundance on the dynamics and trends of 10 vertebrate insectivore species in the UK. We explored three key questions: (1) Are long-term trends and interannual variation between insect and insectivore pairings correlated over space? (2) Does information on insect abundance predict insectivore abundance change? (3) After controlling for shared static and dynamic environmental factors, is there evidence for a direct effect of insects on insectivore dynamics?

Firstly, we found few spatial associations in trends between insects and insectivores. This is despite all bird species and almost all indices of insect abundance showing evidence of declines (this being consistent with other analyses for these taxa; Bell et al. 2020; Brooks et al. 2012; though also dependent on time-series length and analytic method, e.g. Woodward et al. 2020). The only positive associations between trends across space were for the blue tit, great tit, and corn bunting. This suggests that, though the populations of these insectivore species and insects are declining on average, the declines are not necessarily correlated across space. No bat species were declining on average, and, consequently, it is reasonable to infer that, so far and for the specific species groups we investigated, declines in the insect groups tested are not leading to declines in UK bat abundance (although they may still impact population growth rates).

Overall, this first level of associative testing, though lacking control for possible confounds, provides a high-level overview of spatial patterns and finds no conclusive evidence that declines in the insect groups tested are the primary cause of decline for most of the insectivores we assessed. This is plausible as there is evidence that declines in both insects and insectivores result from a combination of several drivers, including land use change, agricultural intensification, and changing climate (Pearce-Higgins and Morris 2023; Wagner et al. 2021a, b). The contrast in spatial patterning in insects and insectivores suggests that some of these drivers of change may be non-overlapping, or at least, the importance of the drivers may vary between the insects and insectivores. Further evaluation of the underlying causes of regional variation in trends for both insects and insectivores, and the generality of such patterns, would therefore be highly valuable.

For those species where we found associations with insect indices, it was necessary to assess if insect abundance was impacting dynamics after controlling for possible confounds. Similarly, even for those species where there were no associations in trends, it is possible that low insect abundance could cause reduced population numbers in particular years and, for the bats, where we found limited evidence of populations declining, it may still be the case that populations may have been higher, and trends more positive, had insect abundance not decreased.

We found that inclusion of information on insects generally improved the predictive skill of the EDMs, but by only small amounts. Model performance also declined at finer spatial scales, suggesting either increased noise or reduced time-series lengths, which reduced our ability to capture dynamics. Generally, when assessing both predictive and statistical criteria, results were mixed and demonstrated scale dependency. Handling variation across scales is challenging as it influences signal-noise relationships alongside coverage of the data across time and space. These issues are also related to the challenge of generating site-level or regional trends given a particular scheme. For example, for butterflies, local trends and abundance indices have been utilised frequently to understand local drivers of population dynamics, but it is challenging to estimate robust bat population trends at scales smaller than the country-level, due to smaller sample sizes.

We next applied panel estimators to handle shared dynamic and static factors influencing populations and isolate the direct (linear) effect of insects on insectivores. As with the EDMs, we found limited conclusive evidence of a direct effect of insect abundance on insectivore populations. Positive links across the majority of scales tested (but not for all model specifications) were only found for blue tit and great tit. We also found evidence of positive links for grey partridge, corn bunting, and noctule, but only at one scale. However, we also found a handful of negative links in either the lag or concurrent insect abundance at least one scale for blue tit, great tit, soprano pipistrelle and serotine. Ignoring data limitations, the occurrence of negative links and the disconnect between the EDM and the panel estimators could suggest that links between insects and insectivores are not simply bottom-up linear effects (insects influence insectivore abundance) but may include a variety of processes. For example, top-down effects, which have been observed to operate in both bird- and bat-insect systems (Beilke and O’Keefe 2023; Holmes et al. 1979), could generate apparent reverse causation that complicates interpretation, and lagged responses from insectivores combined with density dependence in the insect populations might generate apparent negative relationships. While not feasible given this broad survey, close examination of non-linear approaches like EDM, against a variety of plausible top-down and bottom-up causal structures assessed through linear statistical approaches, may help to identify any complex or non-linear associations.

Overall, our analysis of trends and links between insectivores and insect food indices provides some evidence that populations of great tit, blue tit, and grey partridge may be influenced by the abundance of their insect prey. For the blue tit, this link is also consistent with results from Evans et al. (2024), albeit using different methods and different aggregations of the underlying data. For the remaining bird species, we provide limited evidence that the abundance of the insect prey assessed is driving dynamics or declines. These results may be surprising as there is considerable circumstantial evidence that insectivorous birds are impacted by changing insect abundance (Tallamy and Shriver 2021). Evidence from synthesis (Grames et al. 2023) and studies utilising monitoring data (Martay et al. 2023; Yazdanian et al. 2024) also strongly suggest a link between insect abundance and either insectivore vital rates or distributions. None of these studies, however, evaluate or find a direct link between insect abundance and population changes in insectivores per se. Consequently, it may be that limitations with the data (see below) or the complex influence of multiple drivers acting on both insect and insectivores make it challenging to detect direct links between insect and insectivore population change at large scales.

None of the bat species were declining on average, and although we found some evidence (using EDM) that insect abundance provided some predictive information for bats, there was only one positive link using the panel estimators (noctule and Diptera). It is challenging to link bat population trends to trends in their insect prey (though see Langton et al., 2010; Vaughan et al., 1997). Bat populations in the UK are at historic lows, likely due to habitat change (Razgour et al. 2024) and, therefore, changes to insect abundance may not be a main factor limiting populations. Bats are also highly mobile foragers, allowing them to exploit ephemeral concentrations of aerial insects, which might mitigate the effects of reductions in local insect abundances (Fenton, 1997; Stidsholt et al., 2024). In sum, this analysis does not provide evidence that bat populations are limited by the changing abundance of the insect groups tested here, though we identify that generating more robust regional trends for bats and assessing both the potential for top-down and bottom-up dynamics could better clarify links between bat populations and their prey.

While citizen science monitoring data were not originally designed to test direct trophic links, they remain the most extensive and systematically collected datasets available at national scale, and underpin widely accepted species trends and conservation assessments. Our analysis therefore represents one of the only available routes currently available to test insect–insectivore links at broad scales and, although we covered several methodological possibilities in our specification approach, we recognise limitations in our analyses. Principally, the data used to make comparisons between insects and insectivores are not from the same locations and the underlying indices were not designed with the analysis undertaken here in mind. Additionally, there is a mismatch between the life-stages recorded by the schemes and the life-stage consumed by the birds and bats. For example, the blue and great tits principally consume moth larvae, but the number of adults is recorded by the Rothamsted scheme; similarly, the bat species will principally consume adult Diptera, but we utilise data on freshwater larvae. While we expect abundance across life-stages to be correlated, this mismatch does introduce further measurement error.

Future progress in linking insect and insectivore population change, therefore, may depend less on increasing the number of taxa or sites monitored, and more on improving the alignment of insect and insectivore data in space and time (although increases in the number of taxa and sites monitored would expand the power of opportunistic analyses such as this one). Specifically, co-located monitoring that measures insect and insectivore dynamics within the same spatial units and years would substantially reduce ambiguity arising from spatial mismatch. Collecting new data to meet this need remains challenging, however, as the statistical approaches we apply here require multiple sites and years to both separate site-level and year-level responses to environmental variation, and to capture the underlying dynamic behaviour of the populations. Importantly, several of these improvements could be achieved by closer integration of existing monitoring schemes, (e.g. aligning moth traps and bat transects) rather than by entirely new data collection efforts. This would support the schemes main purpose of assessing changes in abundance and occupancy for target species while also offering a realistic pathway toward more tractable inference of trophic interactions. Finally, there is also the potential for methodological improvements to our approach such as developing indices of prey availability that aggregate multiple insect taxa, potentially weighted by biomass or energetic value (Anthony & Kunz, 1977; Naef-Daenzer et al., 1999) and more refined schemes to pair insect and insectivore monitoring sites.

In conclusion, for the species assessed, we find limited evidence that insect declines are currently the primary cause of insectivore declines in the UK. This does not imply absence of effect, but rather highlights the complexity of multiple, sometimes non-overlapping, drivers of biodiversity change. Importantly, our approach demonstrates that an analyst has several tools to robustly assess trophic links in natural systems, but our results point to the need for more targeted, integrated monitoring to assess such links and better inform conservation policy and management.

## Supplementary Information

Below is the link to the electronic supplementary material.

Supplementary Material 1

## Data Availability

Code and data in support of the manuscript is available at 10.5281/zenodo.15037981.

## References

[CR100] Anthony ELP, Kunz TH (1977) Feeding strategies of the little brown bat, Myotis lucifugus, in southern New Hampshire. Ecol 58:775–786. 10.2307/1936213

[CR1] Barlow K, Briggs P, Haysom K, Hutson A, Lechiara N, Racey P, Walsh A, Langton S (2015) Citizen science reveals trends in bat populations: The National Bat Monitoring Programme in Great Britain. Biol Conserv 182:14–26

[CR2] Beilke EA, O’Keefe JM (2023) Bats reduce insect density and defoliation in temperate forests: An exclusion experiment. Ecology, 104(2), e3903 10.1002/ecy.3903 PMC10078224 36310413

[CR3] Bell JR, Blumgart D, Shortall CR (2020) Are insects declining and at what rate? An analysis of standardised, systematic catches of aphid and moth abundances across Great Britain. Insect Conserv Divers 13(2):115–126. 10.1111/icad.12412 32215052 10.1111/icad.12412 PMC7079554

[CR4] Bergé L (2018) Efficient estimation of maximum likelihood models with multiple fixed-effects: The R package FENmlm. *CREA Discussion Papers*, *13*

[CR5] Botham M, Brereton T, Harrower C, Middlebrook I, Roy D B. (2020) United kingdom butterfly monitoring scheme: site indices 2019. NERC Environ Inform Data Centre. 10.5285/180a1c76-bceb-4264-872b-deddfe67b3de

[CR6] Bowler DE, Heldbjerg H, Fox AD, de Jong M, Böhning-Gaese K (2019) Long-term declines of European insectivorous bird populations and potential causes. Conserv Biol 33(5):1120–1130. 10.1111/cobi.13307 30912605 10.1111/cobi.13307

[CR7] Brooks DR, Bater JE, Clark SJ, Monteith DT, Andrews C, Corbett SJ, Beaumont DA, Chapman JW (2012) Large carabid beetle declines in a United Kingdom monitoring network increases evidence for a widespread loss in insect biodiversity. J Appl Ecol 49(5):1009–1019. 10.1111/j.1365-2664.2012.02194.x

[CR8] Byrnes JE, Dee LE (2025) Causal inference with observational data and unobserved confounding variables. Ecol Lett, 28(1), e70023 10.1111/ele.70023 PMC11750058 39836442

[CR9] Chang C-W, Ushio M, Hsieh C (2017) Empirical dynamic modeling for beginners. Ecol Res 32(6):785–796. 10.1007/s11284-017-1469-9

[CR10] Clayton S (2003) Environmental identity: A conceptual and an operational definition. Identity and the Natural Environment: The Psychological Significance of Nature/The MIT Press

[CR11] Cleveland RB, Cleveland WS, McRae JE, Terpenning I (1990) STL: A seasonal-trend decomposition. J off Stat 6(1):3–73

[CR12] De Chaisemartin C, d’Haultfoeuille X (2020) Two-way fixed effects estimators with heterogeneous treatment effects. Am Econ Rev 110(9):2964–2996

[CR13] Dennis EB, Morgan BJT, Freeman SN, Brereton TM, Roy DB (2016) A generalized abundance index for seasonal invertebrates. Biometrics 72(4):1305–1314. 10.1111/biom.12506 27003561 10.1111/biom.12506

[CR14] Didham RK, Basset Y, Collins CM, Leather SR, Littlewood NA, Menz MHM, Müller J, Packer L, Saunders ME, Schönrogge K, Stewart AJA, Yanoviak SP, Hassall C (2020) Interpreting insect declines: Seven challenges and a way forward. Insect Conserv Divers 13(2):103–114. 10.1111/icad.12408

[CR15] Edwards AM, Rogers LA, Holt CA (2024) Explaining empirical dynamic modelling using verbal, graphical and mathematical approaches. Ecol Evol 14(5):e10903. 10.1002/ece3.10903 38751824 10.1002/ece3.10903 PMC11094587

[CR103] Environment Agency. (2020). Ecology and fish data explorer. https://environment.data.gov.uk/ecology-fish/

[CR16] Evans LC, Burgess MD, Potts SG, Kunin WE, Oliver TH (2024) Population links between an insectivorous bird and moths disentangled through national-scale monitoring data. Ecol Lett, 27(1), e14362 10.1111/ele.14362 38253060

[CR107] Fenton MB (1997) Science and the conservation of bats. J Mammal 78(1):1–14. 10.2307/1382639

[CR17] Forister ML, Pelton EM, Black SH (2019) Declines in insect abundance and diversity: We know enough to act now. Conserv Sci Pract, 1(8), e80

[CR18] Goodman-Bacon A (2021) Difference-in-differences with variation in treatment timing. J Econ 225(2):254–277

[CR19] Gould E, Fraser HS, Parker TH, Nakagawa S, Griffith SC, Vesk PA, Fidler F, Hamilton DG, Abbey-Lee RN, Abbott JK (2023) Same data, different analysts: Variation in effect sizes due to analytical decisions in ecology and evolutionary biology. BMC Biol 23(1):35 10.1186/s12915-024-02101-x PMC11804095 39915771

[CR20] Goulson D (2019) The insect apocalypse, and why it matters. Curr Biol 29(19):R967–R971 31593678 10.1016/j.cub.2019.06.069

[CR21] Grames EM, Montgomery GA, Youngflesh C, Tingley MW, Elphick CS (2023) The effect of insect food availability on songbird reproductive success and chick body condition: Evidence from a systematic review and meta-analysis. Ecol Lett 26(4):658–673 36798988 10.1111/ele.14178

[CR102] Granger CWJ (1969) Investigating causal relations by econometric models and cross-spectral methods. Econometrica 37(3):424–438. 10.2307/1912791

[CR22] Hallmann CA, Foppen RP, Van Turnhout CA, De Kroon H, Jongejans E (2014) Declines in insectivorous birds are associated with high neonicotinoid concentrations. Nature 511(7509):341–343 25030173 10.1038/nature13531

[CR23] Hallmann CA, Sorg M, Jongejans E, Siepel H, Hofland N, Schwan H, Stenmans W, Müller A, Sumser H, Hörren T, Goulson D, de Kroon H (2017) More than 75% decline over 27 years in total flying insect biomass in protected areas. PLoS ONE 12(10):e0185809–e0185809. 10.1371/journal.pone.0185809 29045418 10.1371/journal.pone.0185809 PMC5646769

[CR24] Hallmann CA, Zeegers T, van Klink R, Vermeulen R, van Wielink P, Spijkers H, van Deijk J, van Steenis W, Jongejans E (2020) Declining abundance of beetles, moths and caddisflies in the Netherlands. Insect Conserv Divers 13(2):127–139. 10.1111/icad.12377

[CR25] Harrower CA, Bell JR, Blumgart D, Botham MS, Fox R, Isaac NJB, Roy DB, Shortall CR (2020) Moth trends for Britain and Ireland from the Rothamsted Insect Survey light-trap network (1968 to 2016). NERC Environmental Information Data Centre. 10.5285/0a7d65e8-8bc8-46e5-ab72-ee64ed851583

[CR26] Holmes RT, Schultz JC, Nothnagle P (1979) Bird predation on forest insects: An exclosure experiment. Science 206(4417):462–463 17809372 10.1126/science.206.4417.462

[CR27] Kropko J, Kubinec R (2020) Interpretation and identification of within-unit and cross-sectional variation in panel data models. PLoS ONE 15(4):e0231349. 10.1371/journal.pone.0231349 32315338 10.1371/journal.pone.0231349 PMC7173782

[CR28] Martay B, Leech DI, Shortall CR, Bell JR, Thackeray SJ, Hemming DL, Pearce-Higgins JW (2023) Aerial insect biomass, but not phenological mismatch, is associated with chick survival of an insectivorous bird. Ibis 165(3):790–807

[CR29] Massimino D, Baillie SR, Balmer DE, Bashford RI, Gregory RD, Harris SJ, Heywood JJN, Kelly LA, Noble DG, Pearce-Higgins JW, Gillings S (2025) The Breeding Bird Survey of the United Kingdom. Glob Ecol Biogeogr 34(1):e13943. 10.1111/geb.13943

[CR30] Met Office D, Hollis, McCarthy M, Kendon M, Legg T (2023) HadUK-grid gridded climate observations on a 5km grid over the UK, v1.2.0.ceda (1836–2022). [Dataset]. NERC EDS Centre for Environmental Information Data Centre Analysis. 10.5285/abf1a6cf830b4f5385c5d73609df8423

[CR31] Molenaar E, Viechtbauer W, van de Crommenacker J, Kingma SA (2024) Neonicotinoids impact all aspects of bird life: a meta-analysis. Ecol Lett 27(10):e14534. 10.1111/ele.14534 39385588 10.1111/ele.14534

[CR32] Müller J, Hothorn T, Yuan Y, Seibold S, Mitesser O, Rothacher J, Freund J, Wild C, Wolz M, Menzel A (2023) Weather explains the decline and rise of insect biomass over 34 years. Nature 628(8007):349–335. 10.1038/s41586-023-06402-z 37758943 10.1038/s41586-023-06402-z

[CR33] Munch S, Rogers T (2024) GPEDM: Gaussian process regression for empirical dynamic modeling (Version R package version 0.0.0.9008) [Computer software]. https://tanyalrogers.github.io/GPEDM

[CR101] Naef-Daenzer L, Keller LF (1999) The foraging performance of great and blue tits (Parus major and P. caeruleus) in relation to caterpillar development, and its consequences for nestling growth and fledging weight. J Animal Ecol 68:708–718. 10.1046/j.1365-2656.1999.00318.x

[CR34] Oliver T, Roy DB, Hill JK, Brereton T, Thomas CD (2010) Heterogeneous landscapes promote population stability. Ecol Lett 13(4):473–484. 10.1111/j.1461-0248.2010.01441.x 20148927 10.1111/j.1461-0248.2010.01441.x

[CR35] Pearce-Higgins JW, Morris RK (2023) Declines in invertebrates and birds–could they be linked by climate change? Bird Study 69(3–4):59–71

[CR36] Powell KE, Oliver TH, Johns T, González-Suárez M, England J, Roy DB (2023) Abundance trends for river macroinvertebrates vary across taxa, trophic group and river typology. Glob Change Biol 29(5):1282–1295. 10.1111/gcb.16549 10.1111/gcb.16549 PMC10107317 36462155

[CR105] R Core Team (2024) R: A Language and Environment for Statistical Computing. R Foundation for Statistical Computing, Vienna, Austria.

[CR37] Razgour O, Montauban C, Festa F, Whitby D, Juste J, Ibáñez C, Rebelo H, Afonso S, Bekaert M, Jones G (2024) Applying genomic approaches to identify historic population declines in European forest bats. J Appl Ecol 61(1):160–172

[CR38] Rennie S C., Adamson J, Anderson R, Andrews C, Bater J, Bayfield N, Beaton K, Beaumont D, Benham S, Bowmaker M, Wood (2017) UK Environmental Change Network (ECN) carabid beetle data: 1992–2015. NERC Environ Inform Data Centre. 10.5285/8385f864-dd41-410f-b248-028f923cb281

[CR39] Rigal S, Dakos V, Alonso H, Auniņš A, Benkő Z, Brotons L, Chodkiewicz T, Chylarecki P, de Carli E, del Moral JC, Devictor V (2023) Farmland practices are driving bird population decline across Europe. Proc Natl Acad Sci 120(21):e2216573120. 10.1073/pnas.2216573120 37186854 10.1073/pnas.2216573120 PMC10214186

[CR104] Shojaie A, Fox EB (2022) Granger causality: A review and recent advances. Annu Rev Stat Appl 9:289–319. 10.1146/annurev-statistics-040120-010930 10.1146/annurev-statistics-040120-010930 PMC10571505 37840549

[CR40] Simonsohn U, Simmons JP, Nelson LD (2019) Specification curve: Descriptive and inferential statistics on all reasonable specifications. Available SSRN 2694998

[CR41] Stanton R, Morrissey C, Clark R (2018) Analysis of trends and agricultural drivers of farmland bird declines in North America: A review. Agric Ecosyst Environ 254:244–254

[CR108] Stidsholt L, Johnson M, Goerlitz HR, Jakobsen L, Brinkløv S, Madsen P T (2024) Low foraging rates drive large insectivorous bats away from urban areas. Curr Biol 34:1–8. 10.1016/j.cub.2024.01.012 10.1111/gcb.17063 38273536

[CR42] Sugihara G, May R, Ye H, Hsieh C, Deyle E, Fogarty M, Munch S (2012) Detecting causality in complex ecosystems. Science 338(6106):496–500 22997134 10.1126/science.1227079

[CR43] Tallamy DW, Shriver WG (2021) Are declines in insects and insectivorous birds related? Ornithological Appl 123(1):duaa059. 10.1093/ornithapp/duaa059

[CR44] van der Sluijs JP (2020) Insect decline, an emerging global environmental risk. Curr Opin Environ Sustain 46:39–42

[CR106] Vaughan N (1997) The diets of British bats (Chiroptera). Mammal Rev 27(2):77–94. 10.1046/j.1365-2907.1997.00030.x

[CR45] Wagner DL, Fox R, Salcido DM, Dyer LA (2021a) A window to the world of global insect declines: Moth biodiversity trends are complex and heterogeneous. Proceedings of the National Academy of Sciences, 118(2), e2002549117. 10.1073/pnas.2002549117 10.1073/pnas.2002549117 PMC7812805 33431565

[CR46] Wagner DL, Grames EM, Forister ML, Berenbaum MR, Stopak D (2021b) Insect decline in the Anthropocene: Death by a thousand cuts. Proceedings of the National Academy of Sciences, 118(2), e2023989118 10.1073/pnas.2023989118 PMC7812858 33431573

[CR47] Warren MS, Maes D, van Swaay CAM, Goffart P, Van Dyck H, Bourn NAD, Wynhoff I, Hoare D, Ellis S (2021) The decline of butterflies in Europe: Problems, significance, and possible solutions. Proceedings of the National Academy of Sciences, 118(2), e2002551117. 10.1073/pnas.2002551117 10.1073/pnas.2002551117 PMC7812787 33431566

[CR48] Wilson EO (1986) Biophilia. Harvard University Press

[CR49] Woodward I, Massimino D, Hammond M, Barber L, Barimore C, Harris S, Leech D, Noble D, Walker R, Baillie S (2020) BirdTrends 2020: Trends in numbers, breeding success and survival for UK breeding birds. BTO Research Report, *732*

[CR50] Wooldridge JM (2010) Econometric analysis of cross section and panel data. MIT Press

[CR51] Yazdanian M, Kankaanpää T, Merckx T, Huikkonen I-M, Itämies J, Jokimäki J, Lehikoinen A, Leinonen R, Pöyry J, Sihvonen P, Kivelä SM (2024) Evidence for bottom-up effects of moth abundance on forest birds in the north-boreal zone alone. Ecol Lett 27(12):e14467. 10.1111/ele.14467 39739322 10.1111/ele.14467 PMC11686949

