# Supplementary material for: Assessing direct effects of insect change on insectivore populations in the United Kingdom
Source: Biodivers Conserv. 2026 Apr 6;35(5):134. doi: 10.1007/s10531-026-03329-5 (PMC13053585; doi:10.1007/s10531-026-03329-5)
Supplement: Supplementary file 1 — Supplementary Material 1 [file 10531_2026_3329_MOESM1_ESM.docx]

**Assessing direct effects of insect change on insectivore populations in the United Kingdom: Supplementary materials**

| Taxa | 100 | 50 | 10 |
| --- | --- | --- | --- |
| Blue tit | ↘ | ↘ | ↘ |
| Great tit | ↘ | ↘ | ↘ |
| Corn bunting | ↘ | ↘ | ↘ |
| Grey partridge | ↘ | ↘ | ↘ |
| Skylark | ↘ | ↘ | ↘ |
| Common pipistrelle | → | → | → |
| Soprano pipistrelle | → | → | → |
| Noctule | → | → | → |
| Serotine | → | → | → |
| Daubenton's bat | → | → | → |
| Moth index | → | → | ↘ |
| Butterfly index | ↘ | ↘ | ↘ |
| Diptera index | ↘ | ↘ | ↘ |
| Beetle index | ↘ | ↘ | ↘ |

Table S1. Summary of average linear trends for insectivores and insect indices at different grid aggregations after removing the year 2020 to remove possible effects of the covid pandemic on monitoring. Positive trends are indicated by ↗, negative trends are indicated by ↘, and no change is shown by →. Scales without data presented are indicated by -.

Table S2: Associations between trends and the remainder (interannual change) for insectivore-insect pairs after removing the year 2020 to remove possible effects of the covid pandemic on monitoring. Correlations between insectivores and food indices for trends and remainders across scales. The green points represent positive associations, the red points negative associations, and the yellow points uncertain associations.


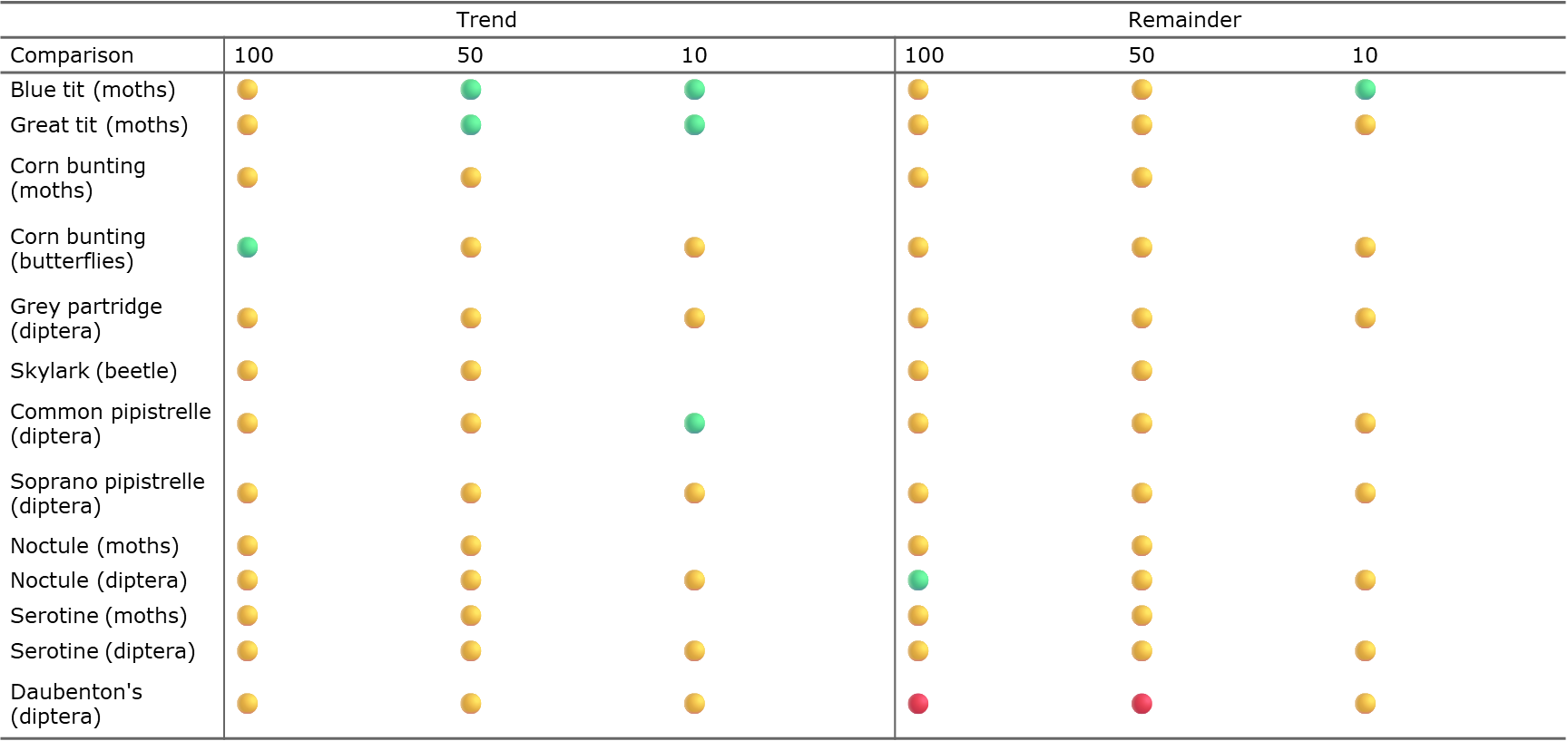


Table S3. The number of comparisons within grid squares across different scales for insect and insectivore pairs.

| Predator index | Insect index | n | grid |
| --- | --- | --- | --- |
| Blue tit | Moth index | 33 | 100 |
| Blue tit | Moth index | 72 | 50 |
| Blue tit | Moth index | 69 | 10 |
| Great tit | Moth index | 32 | 100 |
| Great tit | Moth index | 72 | 50 |
| Great tit | Moth index | 69 | 10 |
| Corn bunting | Moth index | 17 | 100 |
| Corn bunting | Moth index | 24 | 50 |
| Corn bunting | Butterfly index | 19 | 100 |
| Corn bunting | Butterfly index | 34 | 50 |
| Corn bunting | Butterfly index | 21 | 10 |
| Grey partridge | Diptera index | 19 | 100 |
| Grey partridge | Diptera index | 50 | 50 |
| Grey partridge | Diptera index | 31 | 10 |
| Skylark | Beetle index | 8 | 100 |
| Skylark | Beetle index | 10 | 50 |
| Common pipistrelle | Diptera index | 20 | 100 |
| Common pipistrelle | Diptera index | 40 | 50 |
| Common pipistrelle | Diptera index | 20 | 10 |
| Soprano pipistrelle | Diptera index | 18 | 100 |
| Soprano pipistrelle | Diptera index | 30 | 50 |
| Soprano pipistrelle | Diptera index | 10 | 10 |
| Noctule | Moth index | 18 | 100 |
| Noctule | Moth index | 27 | 50 |
| Noctule | Diptera index | 18 | 100 |
| Noctule | Diptera index | 31 | 50 |
| Noctule | Diptera index | 10 | 10 |
| Serotine | Moth index | 9 | 100 |
| Serotine | Moth index | 13 | 50 |
| Serotine | Diptera index | 10 | 100 |
| Serotine | Diptera index | 17 | 50 |
| Serotine | Diptera index | 5 | 10 |
| Daubenton's | Diptera index | 19 | 100 |
| Daubenton's | Diptera index | 48 | 50 |
| Daubenton's | Diptera index | 37 | 10 |


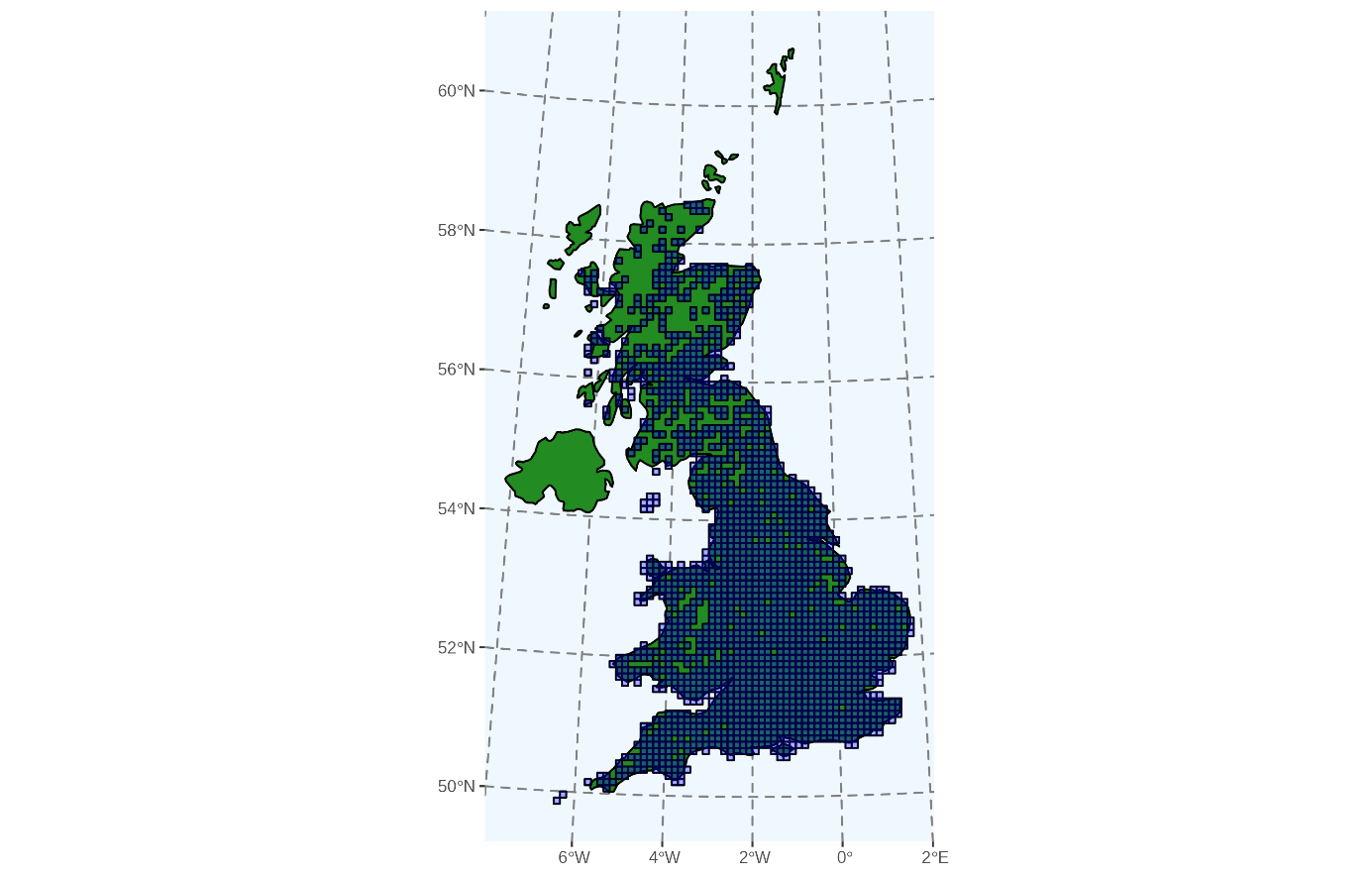


Figure S1. Map of 10km grid squares with observations of great tit abundance.


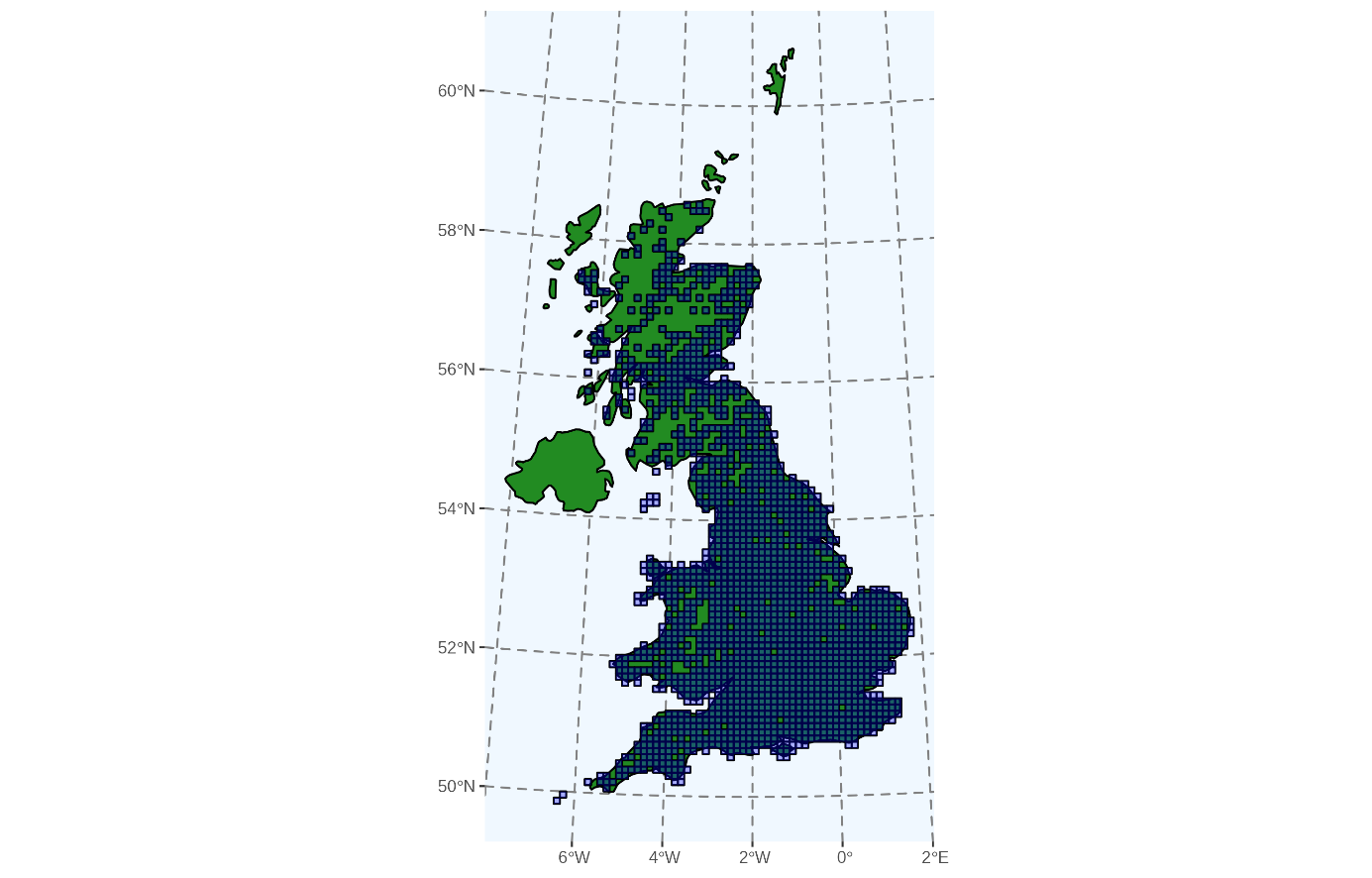


Figure S1. Map of 10km grid squares with observations of blue tit abundance.


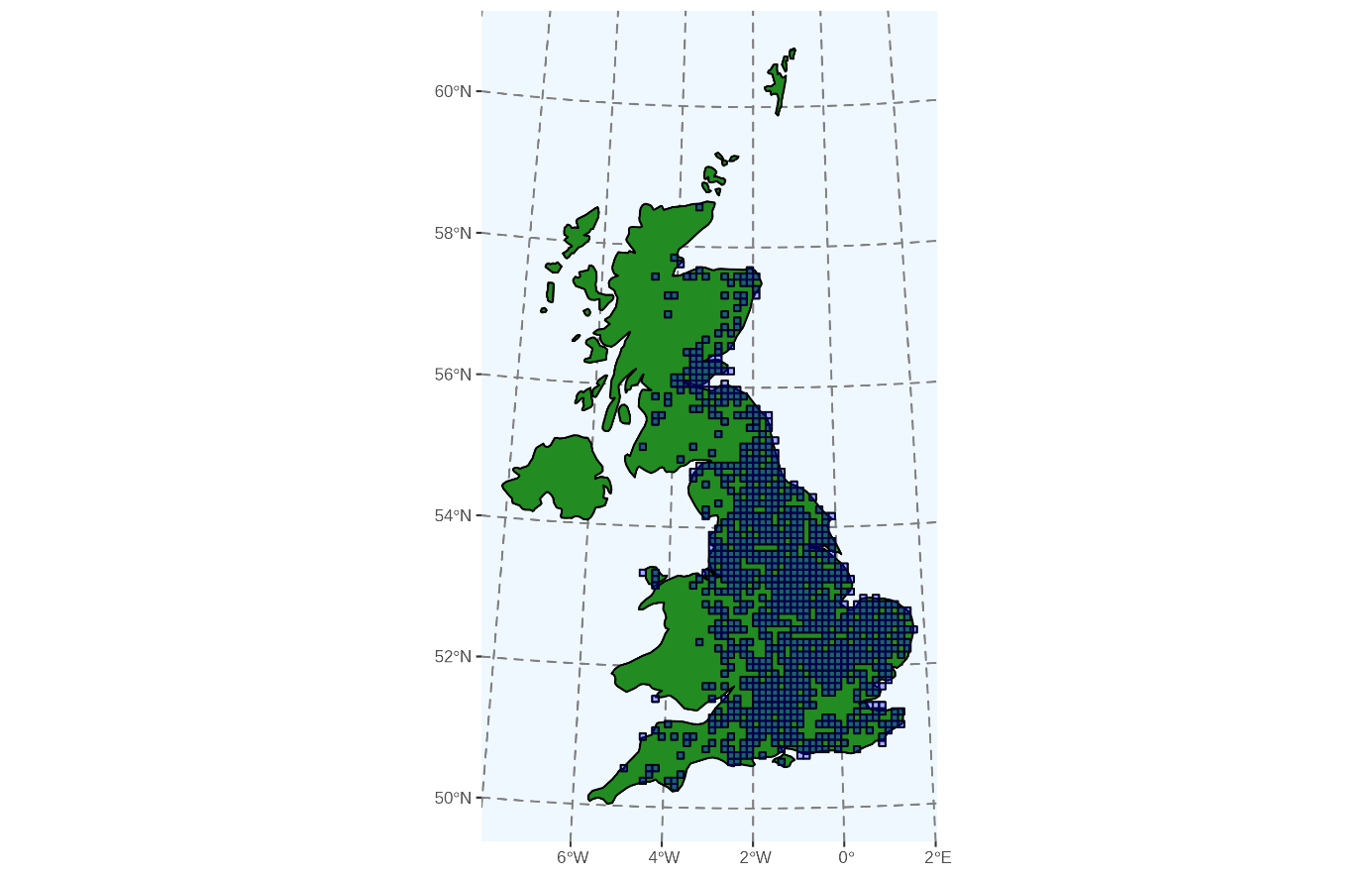


Figure S3. Map of 10km grid squares with observations of grey partridge abundance.


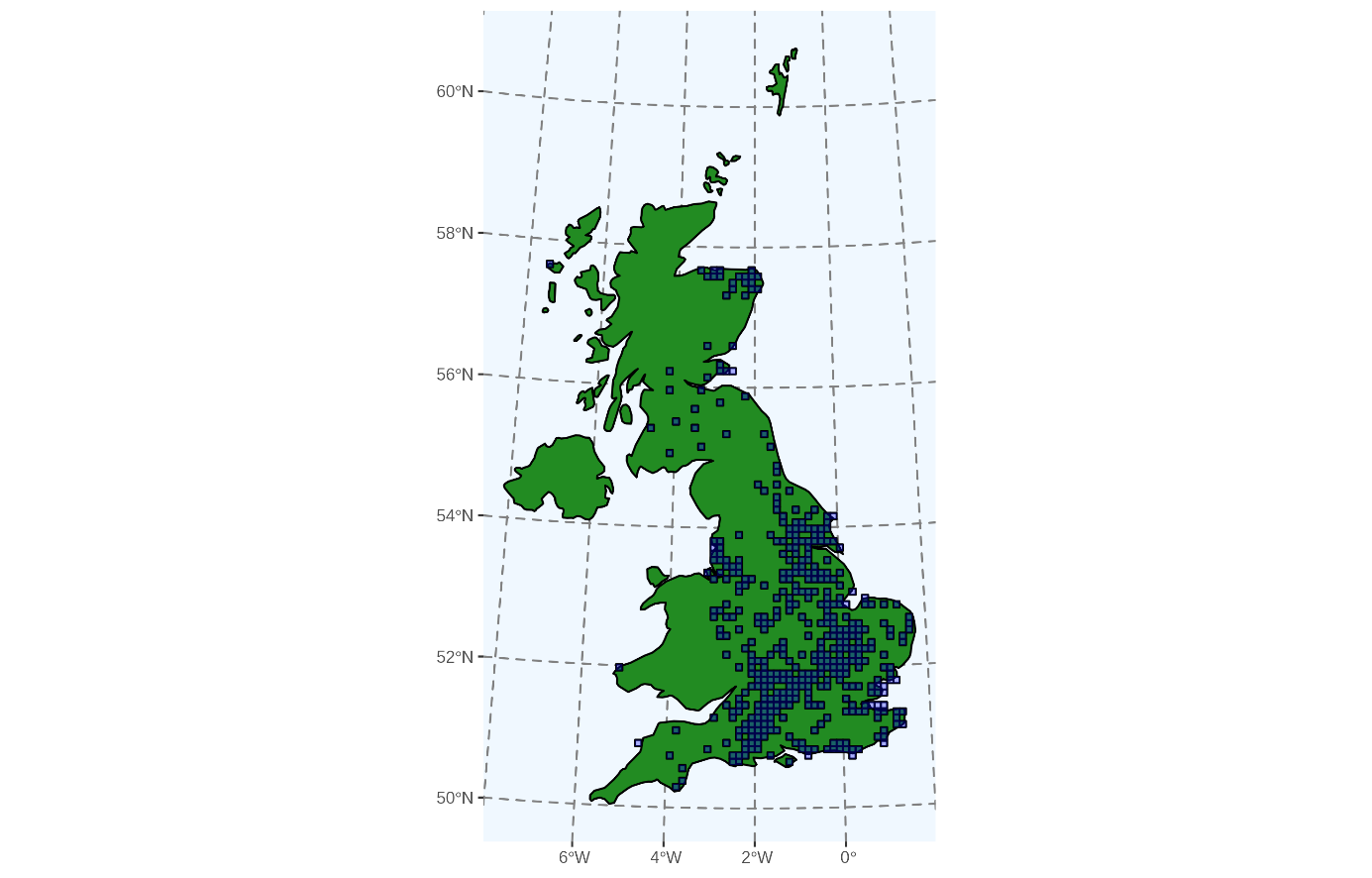


Figure S4. Map of 10km grid squares with observations of corn bunting abundance.


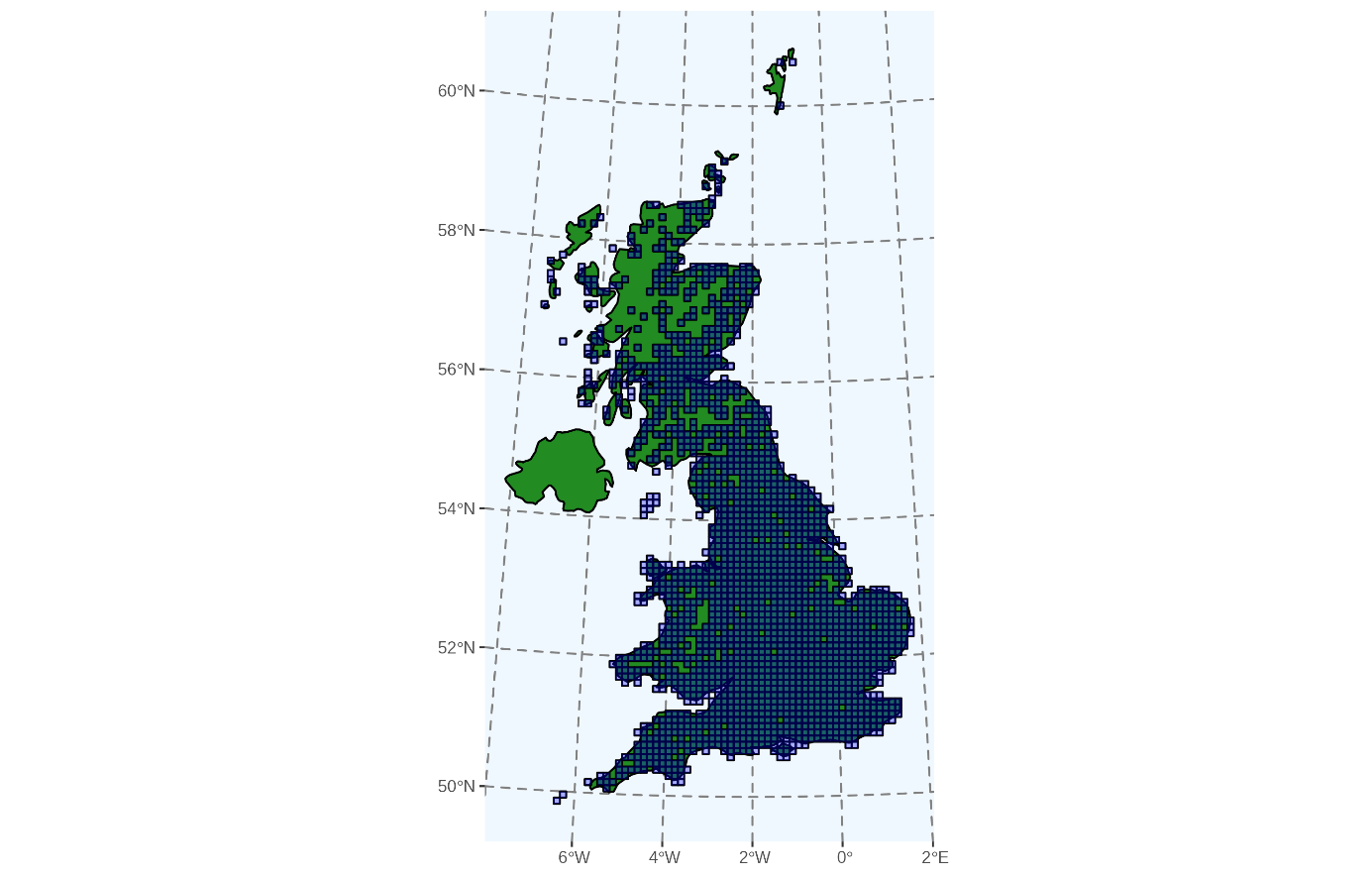


Figure S5. Map of 10km grid squares with observations of skylark abundance.


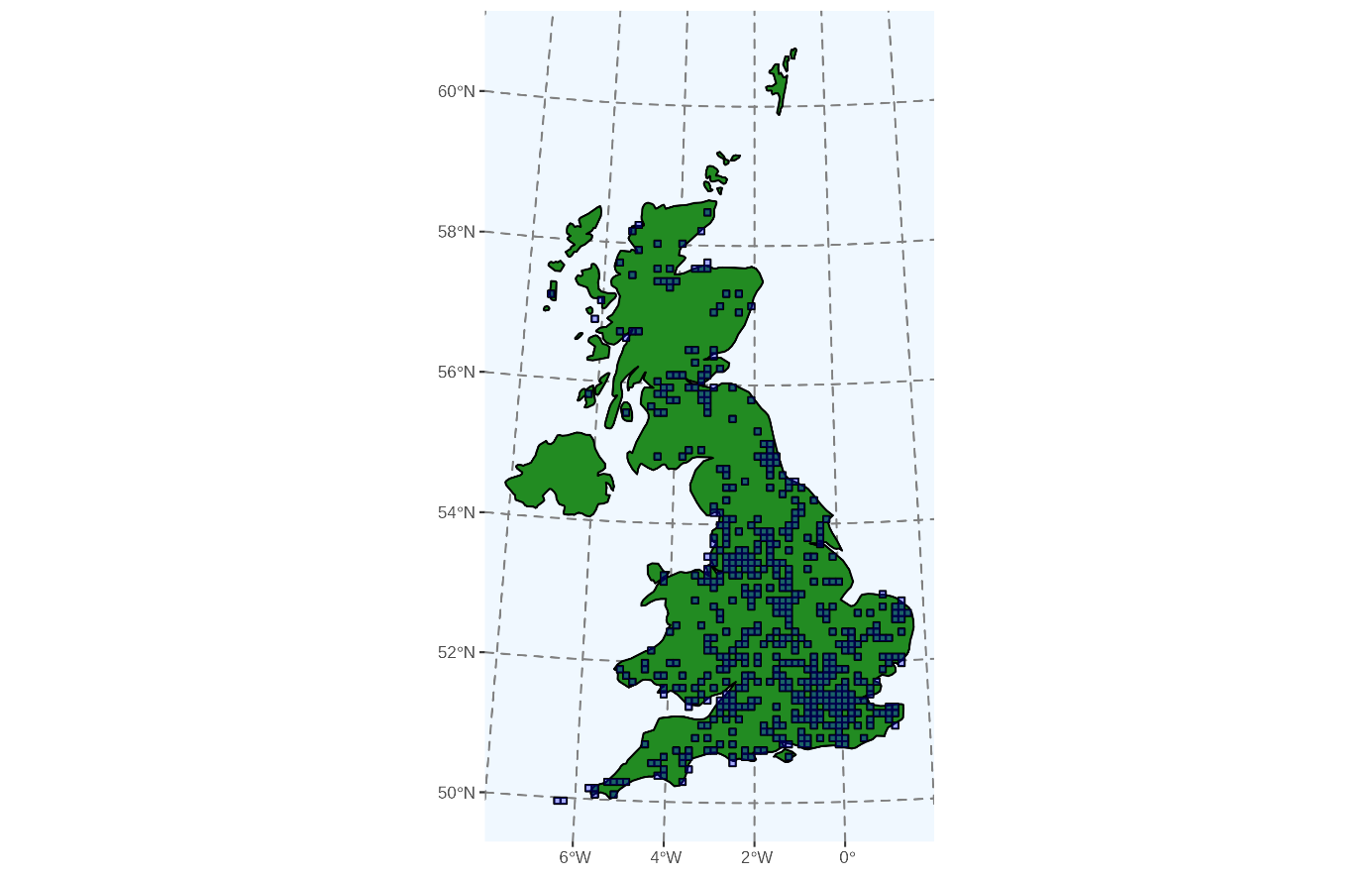


Figure S6. Map of 10km grid squares with observations of common pipistrelle abundance.


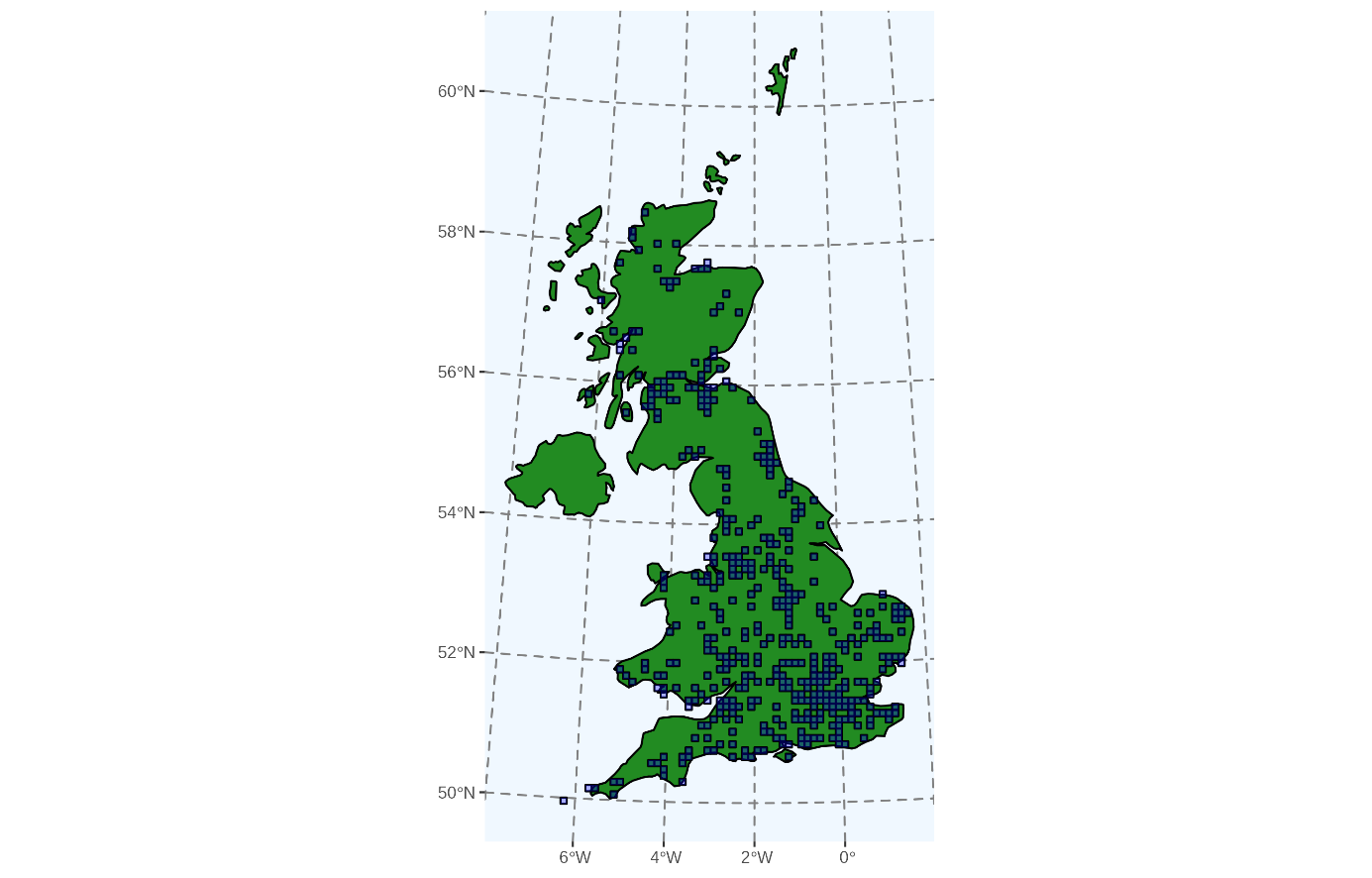


Figure S7. Map of 10km grid squares with observations of soprano pipistrelle abundance.


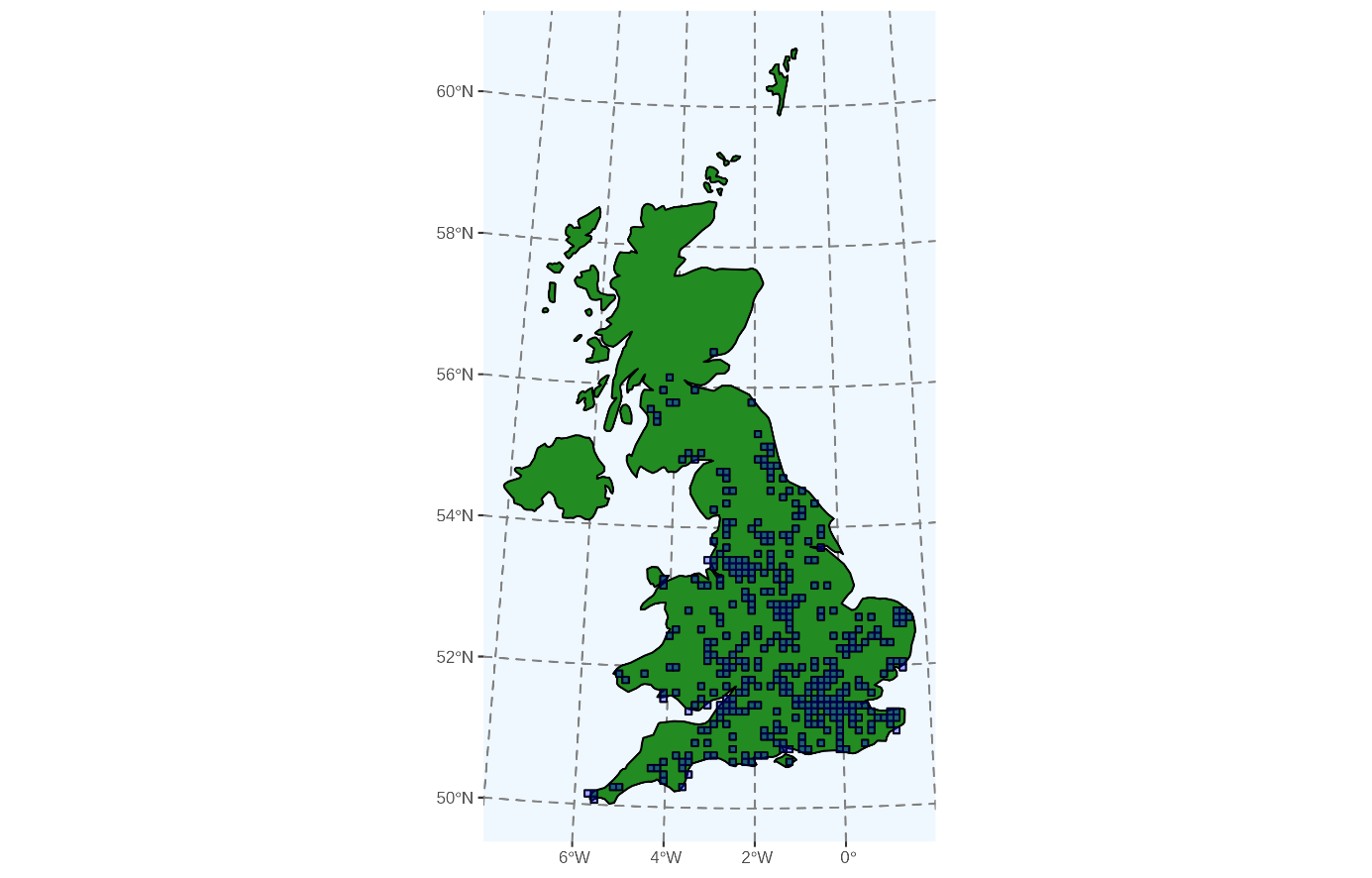


Figure S8. Map of 10km grid squares with observations of noctule abundance.


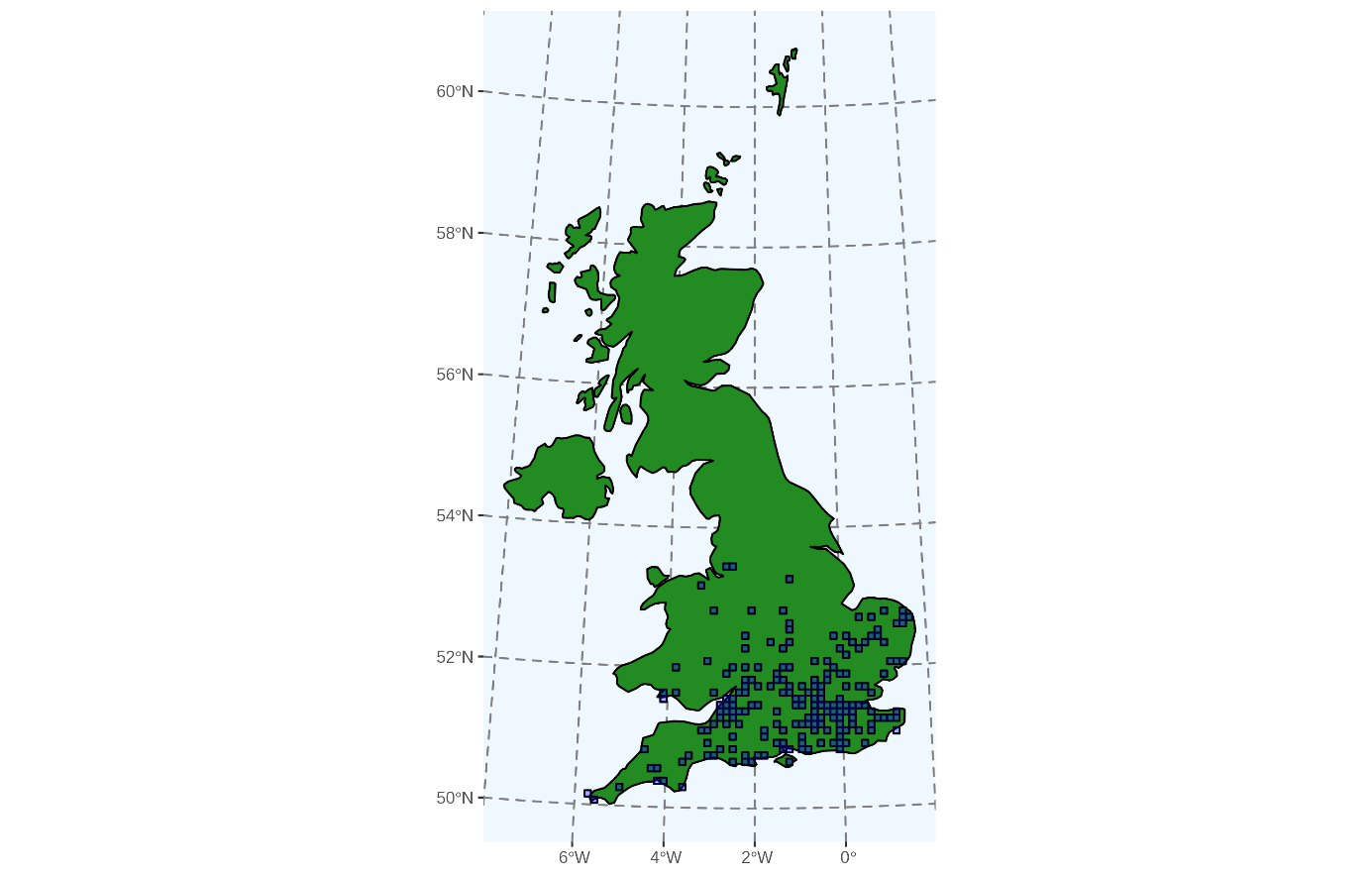


Figure S9. Map of 10km grid squares with observations of serotine abundance.


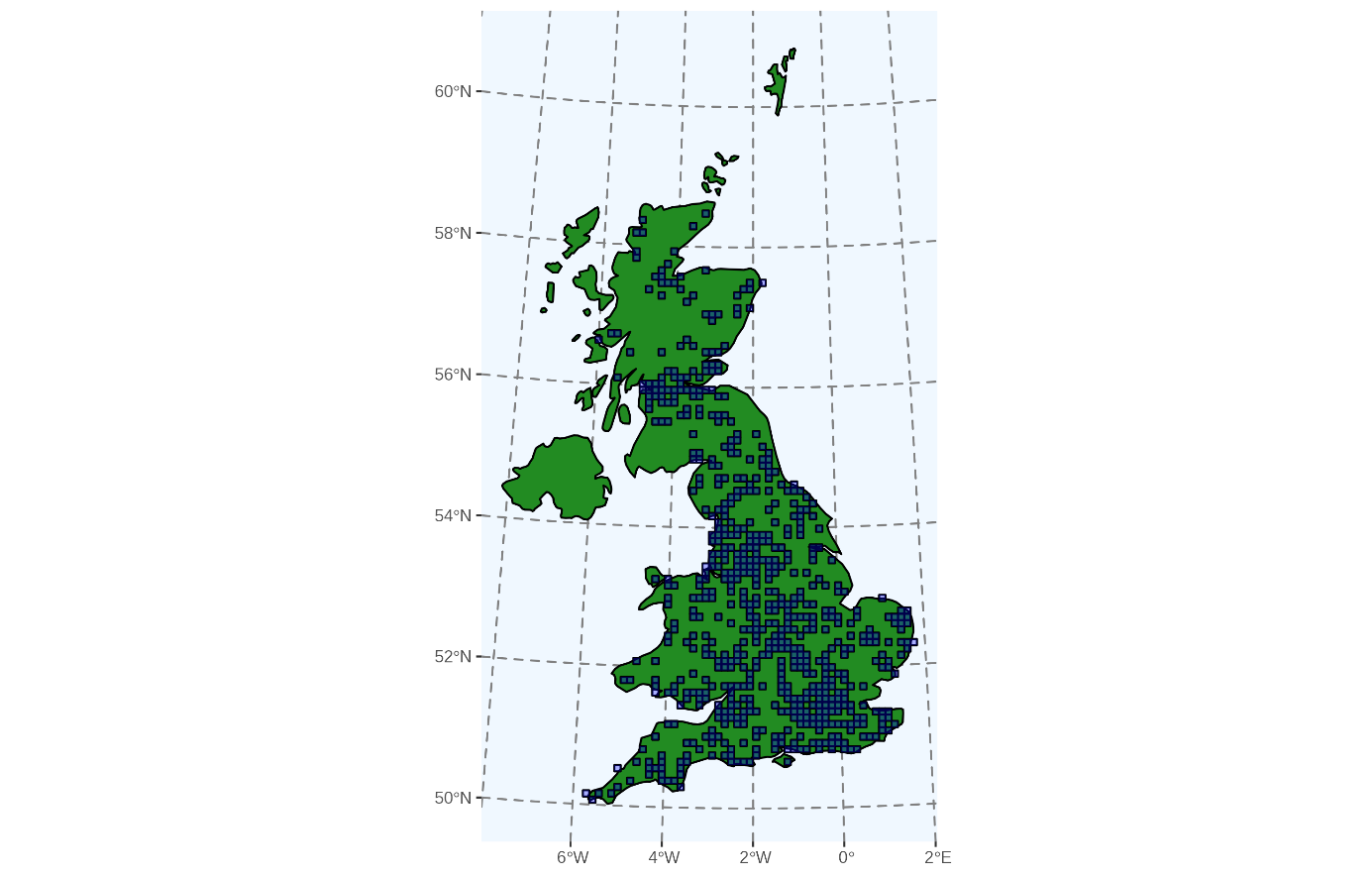


Figure S10. Map of 10km grid squares with observations of daubenton’s bat abundance.


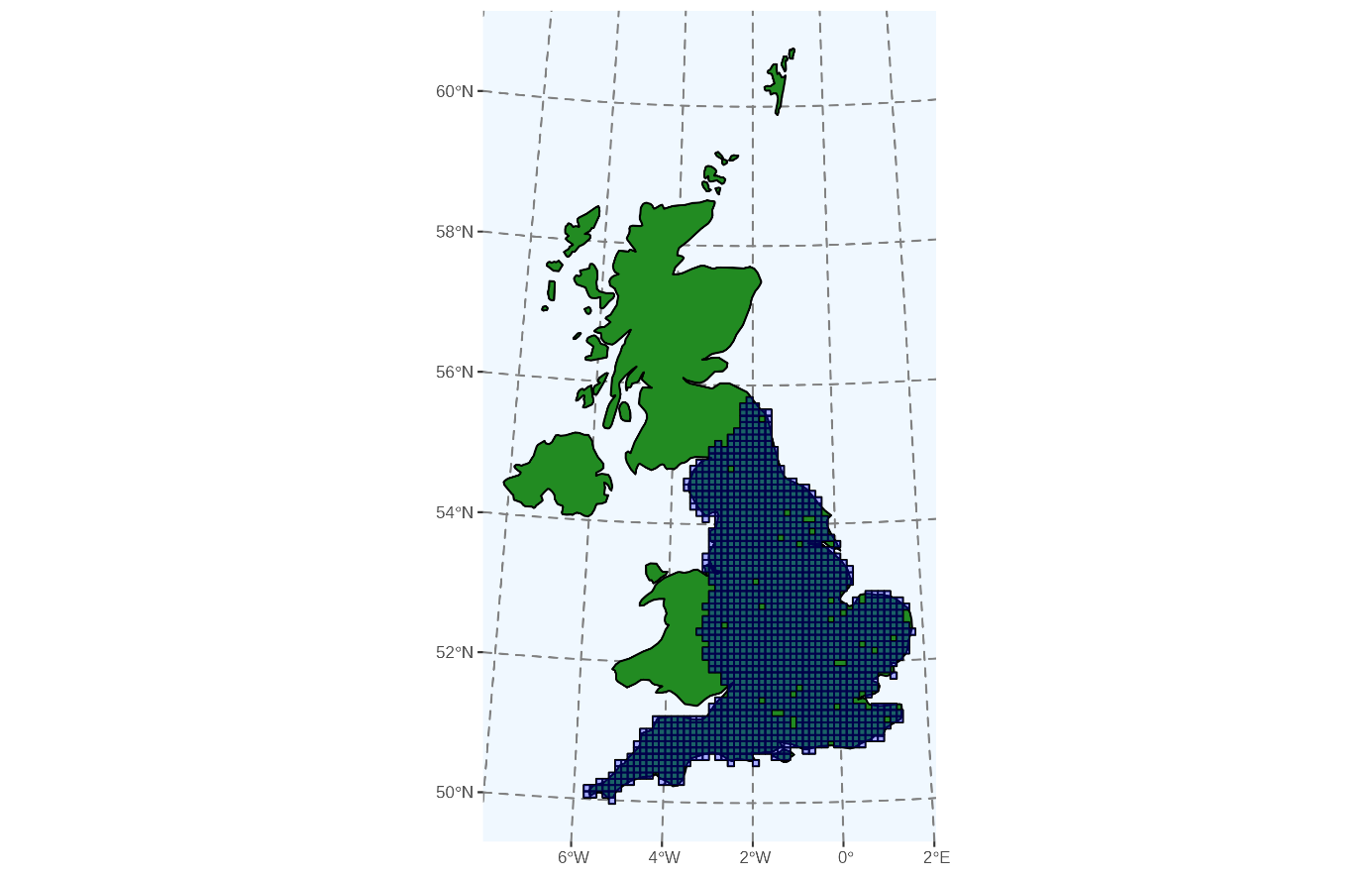


Figure S11. Map of 10km grid squares with observations of freshwater insect abundance.


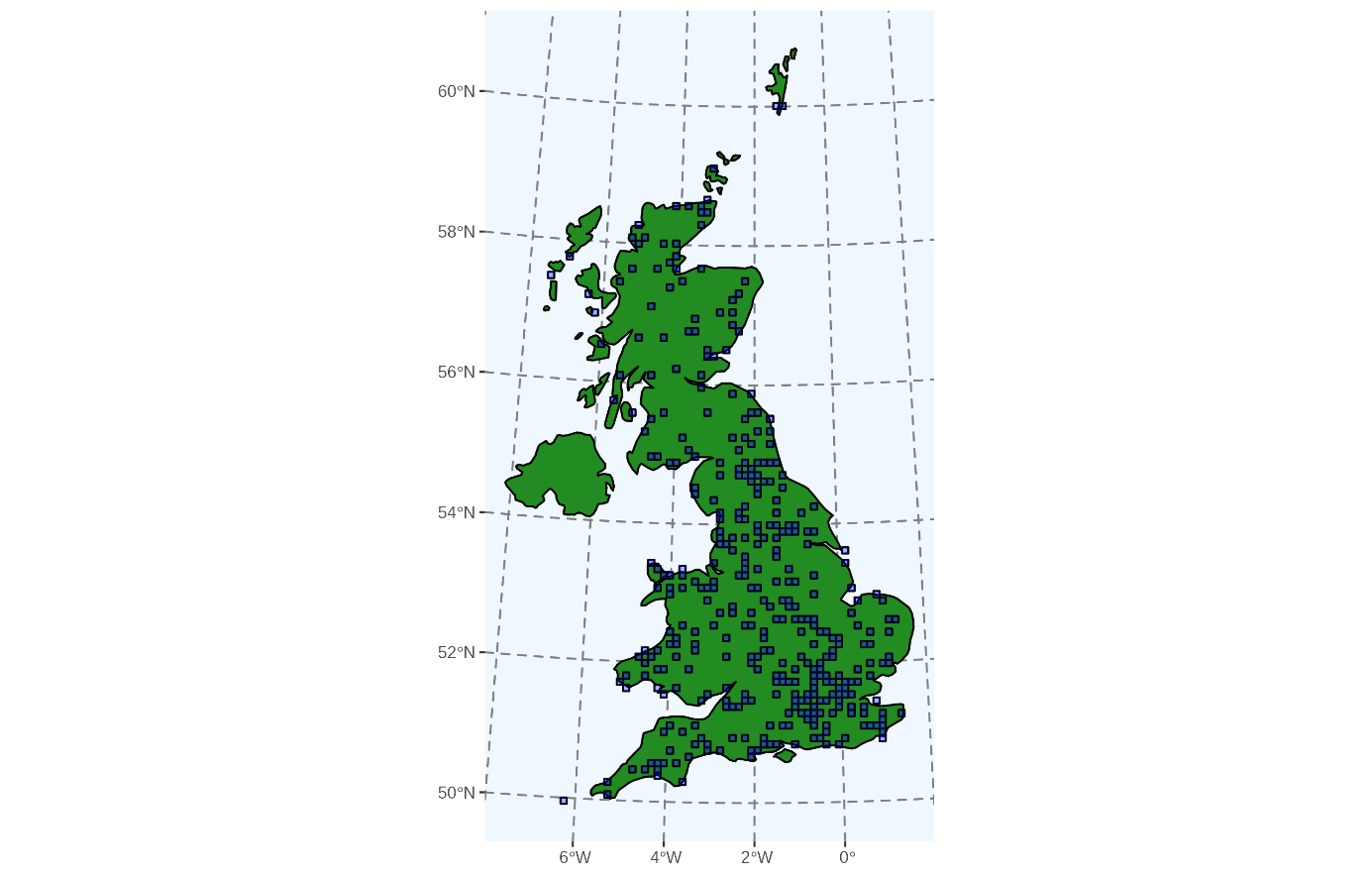


Figure S12. Map of 10km grid squares with observations of moth abundance.


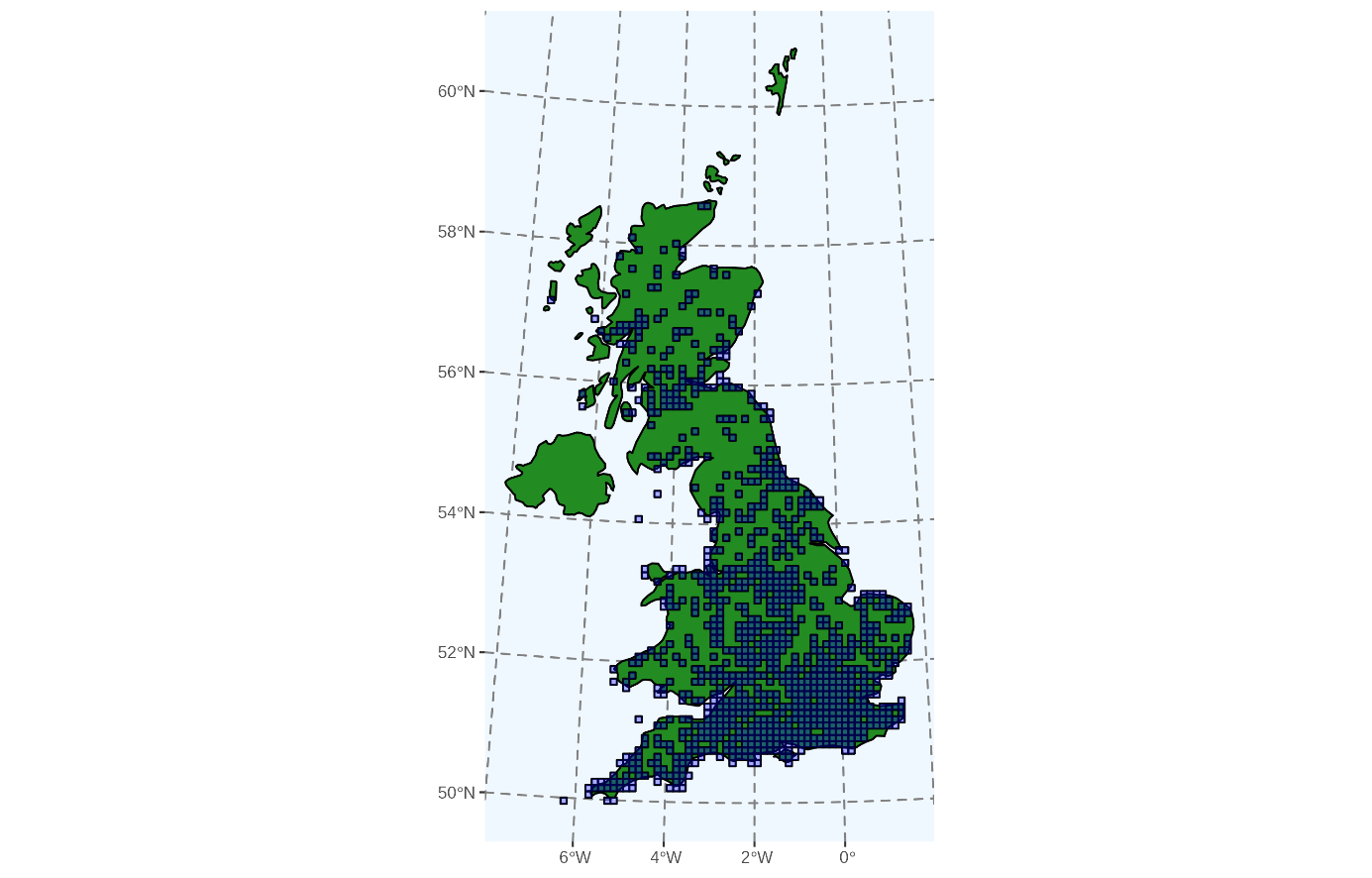


Figure S13. Map of 10km grid squares with observations of butterfly abundance.


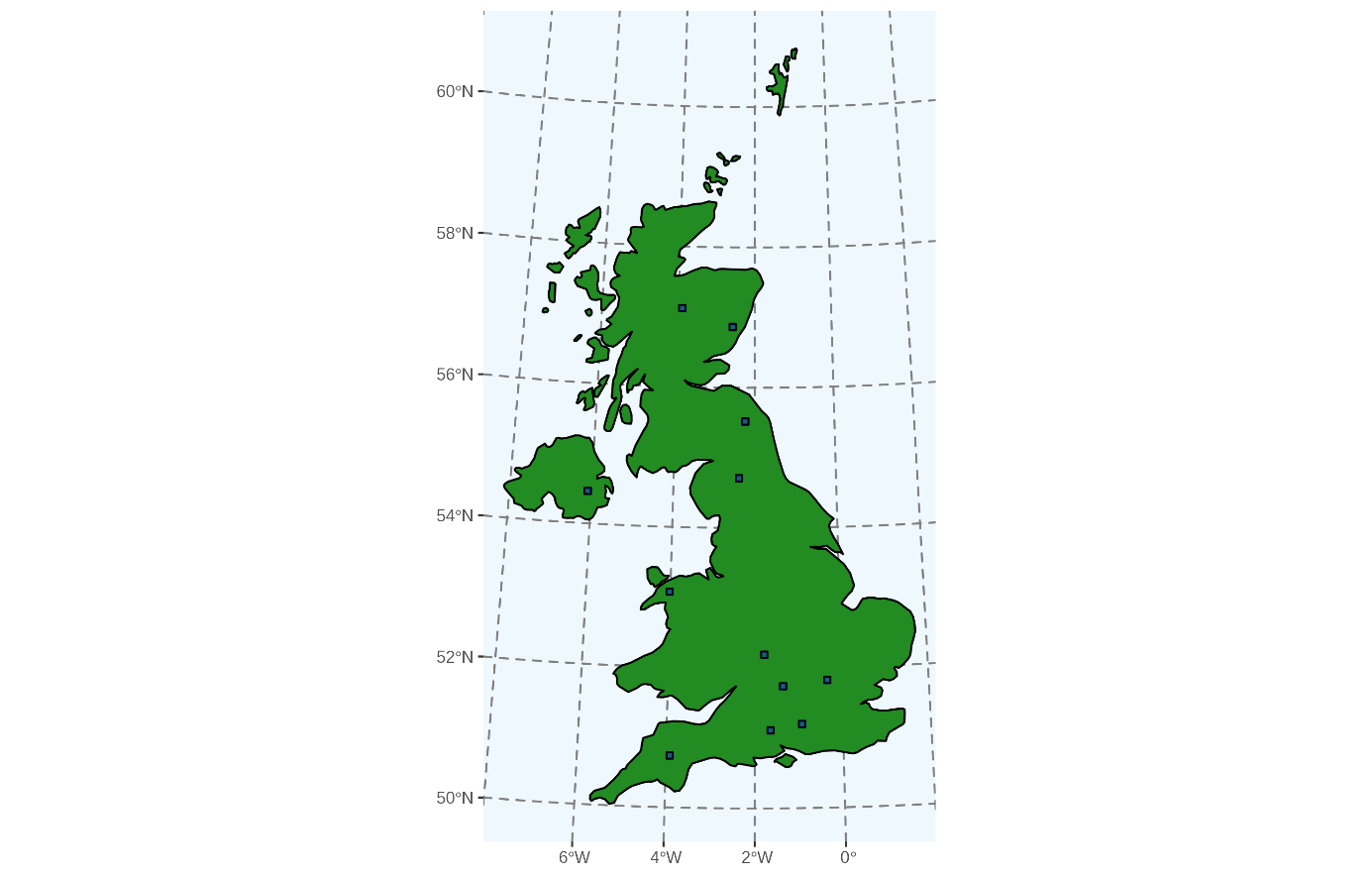


Figure S14. Map of 10km grid squares with observations of beetle abundance.
